# From Molecule to Aggregate: Designing AIE Nanocrystals for Low‐Power Backward Third‐Harmonic Generation Angiography

**DOI:** 10.1002/adma.202414419

**Published:** 2025-04-01

**Authors:** Lidong Du, Hanchen Shen, Changhuo Xu, Xinyan Zhu, Bingnan Wang, Qingqing Zhou, Chunxi Liu, Herman H. Y. Sung, Ryan T. K. Kwok, Jacky W. Y. Lam, Quan Zhou, Tzu‐Ming Liu, Ben Zhong Tang

**Affiliations:** ^1^ MOE Frontiers Science Center for Precision Oncology University of Macau Macau 999078 China; ^2^ Faculty of Health Sciences University of Macau Macau 999078 China; ^3^ Department of Chemistry Hong Kong Branch of Chinese National Engineering Research Center for Tissue Restoration and Reconstruction Division of Life Science State Key Laboratory of Molecular Neuroscience Guangdong‐Hong Kong‐Macau Joint Laboratory of Optoelectronic and Magnetic Functional Materials The Hong Kong University of Science and Technology Clear Water Bay, Kowloon Hong Kong 999077 China; ^4^ Department of Cell Biology Department of Gastroenterology of the Fourth Affiliated Hospital Zhejiang University School of Medicine Hangzhou Zhejiang 310058 China; ^5^ School of Science and Engineering Shenzhen Institute of Aggregate Science and Technology The Chinese University of Hong Kong (CUHK‐Shenzhen) Shenzhen Guangdong 518172 China

**Keywords:** backward third‐harmonic generation, nanocrystallization, resonance enhancement, second near‐infrared excitation, size optimization

## Abstract

Organic materials featuring third harmonic generation (THG) hold great promise for deep‐tissue bioimaging due to their good biocompatibility and second near‐infrared excitation. However, minimizing photodamage from the incident light necessitates significant improvements in the third‐order nonlinear susceptibility. Herein, an organic luminogen called OTBP is developed as a backward THG (BTHG) contrast agent for second near‐infrared (NIR‐II) angiography. OTBP's intense absorption at 433 nm resonantly enhances its BTHG efficiency when excited by a 1300 nm femtosecond laser. In the aggregate state, the robust intermolecular interactions among OTBP molecules realize excellent crystallinity and the facile preparation of nanocrystals (NCs) with a high refractive index of 1.78. By leveraging Mie scattering theory, the best size of OTBP NCs for BTHG collection is attained. These integrated properties result in a high BTHG efficiency of OTBP NCs. Encapsulating the NCs with F‐127 enables ultralow‐power but high‐contrast 3D vasculature imaging with negligible photodamage and background interference. Further elevating the laser power to 60 mW enables the visualization of microvessels at 500 µm with a high SNR of 143. This study offers insights into material design strategies toward efficient organic BTHG contrast agents and paves the way for the materials‐oriented non‐linear optics.

## Graphical abstract

An organic BTHG agent called OTBP with prominent crystallinity is developed. The resonant absorption at 433 nm and prominent crystallinity of OTBP enhance its BTHG efficiency when excited by a 1300 nm femtosecond laser. Tuning the size of OTBP nanocrystals based on Mie scattering theory successfully enables them to achieve high‐contrast muti‐organ angiography with ultra‐low pulse energy (100 pJ) and thereby negligible photodamage risk.

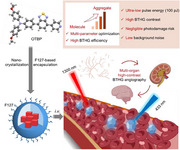

## 1 Introduction

The research paradigm of molecular science canonizes that a macroscopic substance is reducible to a microscopic molecule inheriting all the natures from its parental substance. Indeed, it has been thriving for centuries and has guided the discovery of basic scientific laws for isolated molecular constituents.^[^ ^1^ ^]^ An aggregate, however, is a complex assembly of interactive elementary components in the mesoscopic realm and could retain emergent properties absent from its building blocks.^[^ ^2^, ^3^ ^]^ These aggregate‐state properties are intricately governed by various factors beyond molecular structures, including quantity, geometry, morphology, and interaction at the mesoscale.^[^ ^3^ ^]^ Aggregation‐induced emission (AIE) is a superb representation that reveals how new properties can emerge during the aggregation process and how they can be used to implement extensive applications.^[^ ^4^, ^5^ ^]^ AIE luminogens (AIEgens) do not emit or weakly emit light as single molecules due to their free molecular motions but exhibit bright emission upon aggregation or otherwise spatial confinement to restrict their molecular motions.^[^ ^6^, ^7^ ^]^ Aggregate science is growing fast nowadays and breeding several additional new findings due to the great diversity and complexity of mesoscopic aggregates. For example, recent studies have shown that molecular aggregation can affect and manipulate nonlinear optical (NLO) properties.^[^ ^8^, ^9^, ^10^ ^]^

NLO phenomena have been discovered in a wide range of inorganic and organic materials, and their applications have greatly impacted our daily lives.^[^ ^11^, ^12^, ^13^ ^]^ Third‐harmonic generation (THG) is a third‐order NLO phenomenon, where an intense laser field excites a substance and generates a nonlinear polarization with a tripled optical frequency (one‐third the fundamental wavelength).^[^ ^14^, ^15^, ^16^ ^]^ As third‐order nonlinear susceptibility *χ* ^(3)^ is non‐zero in all matters without any limitation on structural symmetry, THG contrast can reveal more biomedical structures than second harmonic generation (SHG).^[^ ^17^, ^18^, ^19^ ^]^ Due to the ability of THG excitation wavelength to extend into the second near‐infrared window (NIR‐II, 1000–1700 nm) and its high excitation confinement, THG microscopy can achieve deep‐tissue virtual optical biopsies while maintaining high spatial resolution of 0.5 µm,^[^ ^20^, ^21^, ^22^ ^]^ surpassing other modalities, such as photoacoustic imaging (10–50 µm).^[^ ^23^ ^]^ Moreover, THG can be enhanced at a heterogeneous interface where the Gouy‐phase‐shift cancellation is greatly mitigated.^[^ ^24^, ^25^, ^26^ ^]^ Based on the sensitivity to the interface of biomolecular aggregates,^[^ ^21^ ^]^ THG plays an essential role in the label‐free imaging of lipids, cell membranes, or nerve contours.^[^ ^27^ ^]^ Despite these advantages, THG signal collection for bioimaging is still practically challenging. Due to the momentum conservation, the intensity of the forward THG (FTHG) signal is two orders of magnitude higher than that of the BTHG signal.^[^ ^28^ ^]^ However, an epi‐collection scheme is required when imaging optically thick samples. Simply increasing the laser power to achieve a satisfactory BTHG output can cause irreversible photodamage to the tissue.^[^ ^29^ ^]^ Previous research suggested that a safe incident power at the sample is no more than 20 mW for multiphoton imaging. To avoid noticeable photodamage, scientists have to reduce the excitation power at the cost of poor contrast and lengthened acquisition time,^[^ ^29^, ^30^ ^]^ which pose a challenge in obtaining high‐quality and high‐speed THG images in vivo.

Materials with high *χ* ^(3)^ are highly desired to enable a safe excitation laser power when carrying out THG angiography. The refractive index *n* is one of the most critical factors for affording a large *χ* ^(3)^, as described by the following semiempirical Equation (1):^[^ ^31^ ^]^

(1) $$\chi^{\left(3\right)}=\frac{A}{{\left(4\pi \right)}^{4}}{\left(n^{2}-1\right)}^{4}$$

where A is a constant. This relationship is also true at the materials interface, where a larger *n* difference can generate a stronger BTHG signal.^[^ ^32^ ^]^ Inorganic crystals typically exhibit a high *n* and have been adopted as contrast agents for BTHG bioimaging.^[^ ^24^, ^33^, ^34^, ^35^ ^]^ However, due to their poor biocompatibility and potential toxicity, they are not optimal candidates for in vivo applications.^[^ ^36^, ^37^, ^38^, ^39^ ^]^ In contrast, organic small molecules show better biosafety. However, they also encounter difficulties in forming condensed interfaces with high *n* in the aqueous phase. We believe AIEgens are promising candidates to address these issues for the following reasons. First, red‐emissive AIEgens usually feature *π*‐electron conjugation and donor–acceptor (D–A) structures for efficient electronic transitions. Second, conjugated structures of AIEgens bring intense absorption bands as an extra benefit for BTHG enhancement. Once the real electronic transition of organic fluorophores matches the virtual excited state of a BTHG process, the resonance enhancement (RE) effect can alter *χ* ^(3)^ to increase the BTHG efficiency.^[^ ^40^, ^41^, ^42^ ^]^ The RE factor can be more than one order of magnitude in some cases.^[^ ^43^ ^]^ Third, some AIEgens can orderly assemble into mesoscopic aggregates with high crystallinity to ensure high *n* interfaces.^[^ ^44^, ^45^, ^46^ ^]^

**Figure 1:**
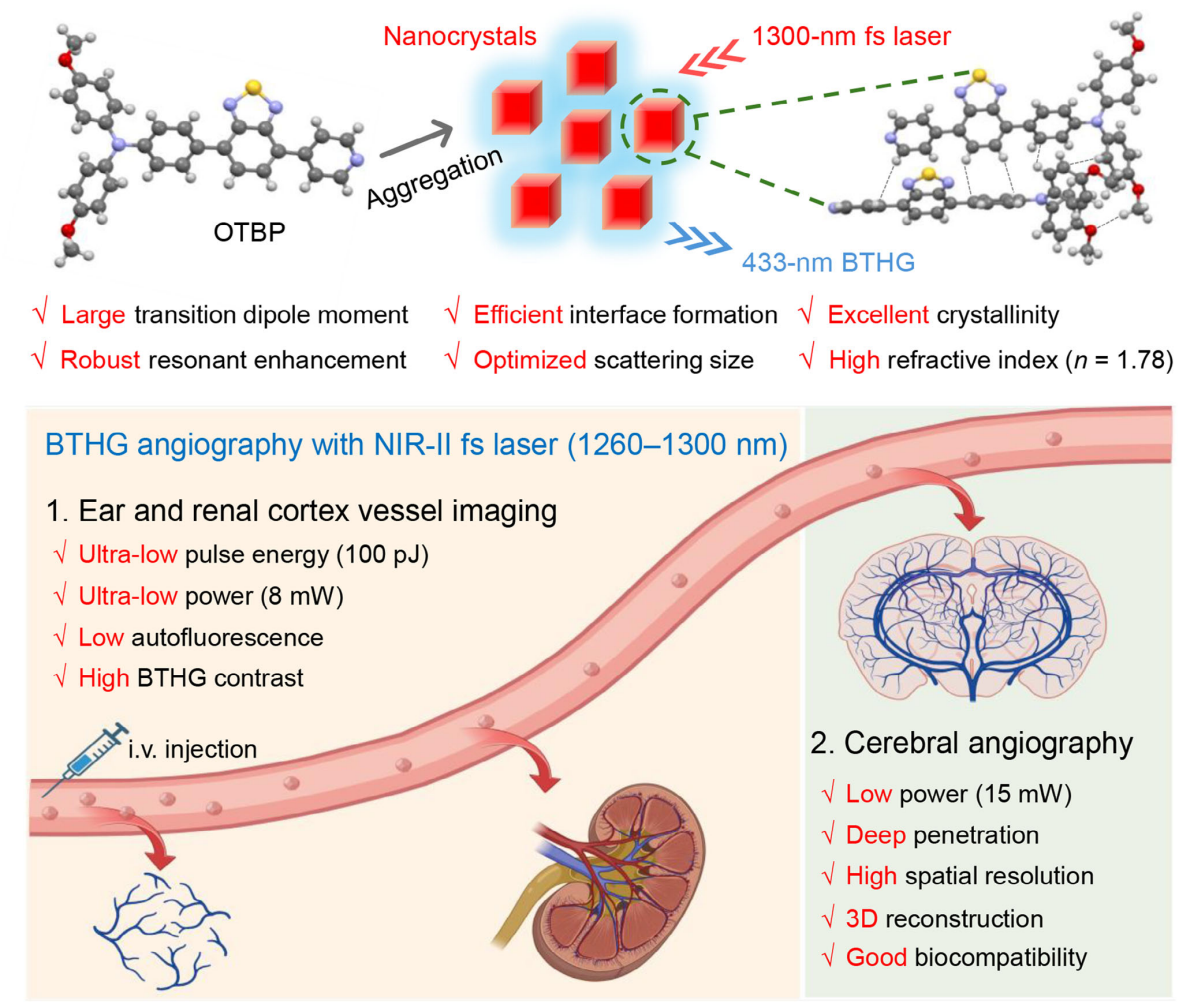
Schematic illustration demonstrating the unique properties of OTBP at the molecular and aggregate levels and low‐power multi‐organ BTHG angiography by using F‐127‐wrapped OTBP nanocrystals.

In this contribution, we have designed D–A structured AIEgens namely 4‐methoxy‐*N*‐(4‐methoxyphenyl)‐*N*‐(4‐(7‐(pyridin‐4‐yl)benzo[c][1,2,5]thiadiazol‐4‐yl)phenyl)aniline (OTBP) and *N*,*N*‐bis(4‐methoxyphenyl)‐7‐(pyridin‐4‐yl)benzo[c][1,2,5]thiadiazol‐4‐amine (ODBP). In terms of molecular structure, OTBP shows better *π*‐electron conjugation and a larger transition dipole moment than ODBP. Besides, the charge transfer absorption band of OTBP is closer to the RE wavelength of 1300 nm‐excited THG compared to that of ODBP, thereby contributing to its stronger resonant BTHG enhancement. These photophysical properties of the molecules work synergistically to provide OTBP with a strong BTHG efficiency. In addition, OTBP can readily form nanoaggregates with superior crystallinity, which can promote interface formation in the aqueous phase. The crystalline film of OTBP exhibits a high *n* of 1.78, and the crystalline nature of OTBP ensures a higher degree of restriction of intramolecular motions to stabilize the virtual electronic state for an efficient BTHG process.^[^ ^47^ ^]^ To further increase the BTHG yield, we adjusted the size of OTBP nanocrystals (NCs) for the optimal Mie scattering cross section at a fixed incident wavelength of 1300 nm, thereby significantly improving the collection efficiency of BTHG signal. These optimizations reduce the required laser power to an ultra‐low level, minimize the photodamage, and achieve high‐contrast multi‐organ angiography without background interference (**Figure** **1**). Furthermore, a 1260 nm laser with higher excitation power is successfully applied to achieve deep‐brain imaging with high SBR. Importantly, OTBP NCs demonstrate very low systematic toxicity, making it a biocompatible contrast agent for BTHG angiography.

## 2 Results and Discussion

### 2.1 Molecular Design for Enhanced BTHG Efficiency

**Figure 2:**
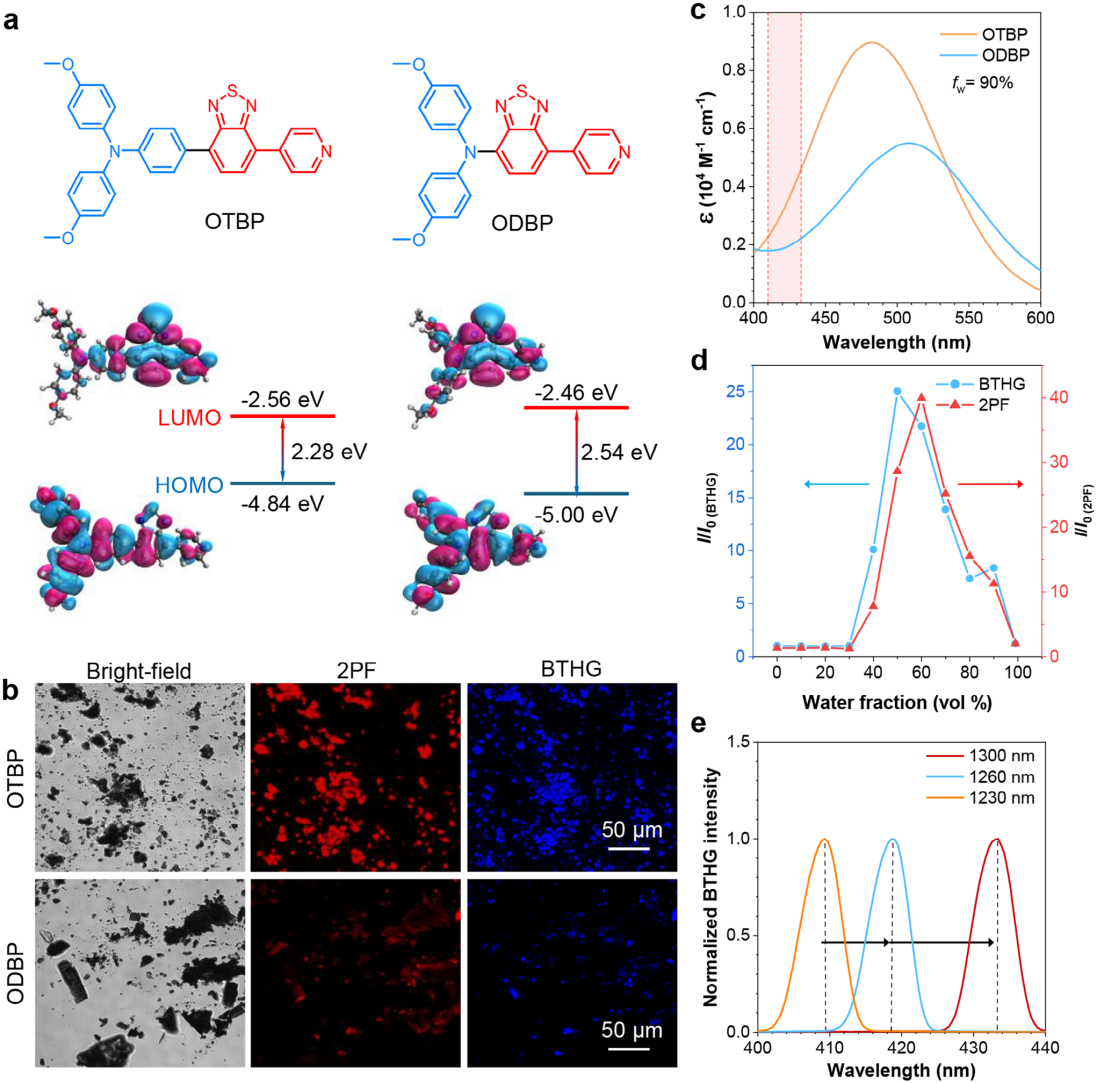
a) Chemical structures of OTBP and ODBP and DFT calculations for their frontier molecular orbitals. b) Bright‐field, 2PF (*λ*_ex_ = 1040 nm, *λ*_em_ = 570–750 nm), and BTHG (*λ*_ex_ = 1300 nm, *λ*_em_ = 425–475 nm) imaging of pristine powders of OTBP and ODBP. c) Absorption spectra of OTBP and ODBP in the DMSO/water mixture with a water volume fraction (*f*_w_) of 90%. [OTBP] = [ODBP] = 10 µm. d) Plots of relative 2PF and BTHG intensity (*I/I*_0_) of OTBP versus *f*_w_ in the DMSO/water mixtures, where *I*_0_ means the signal intensity in DMSO. e) BTHG spectra of OTBP at different incident wavelengths (*λ*_ex_ = 1230, 1260, and 1300 nm).

We designed and synthesized OTBP and ODBP, whose structures were well characterized through ^1^H NMR, ^13^C NMR, and ESI‐TOF‐MS (Figures S1 and S2, Supporting Information). Both molecules feature D–A structures with benzothiadiazole and pyridine moieties as the acceptor (**Figure** **2**a). The blue part stands for the donor, while the red part represents the acceptor. Density functional theory (DFT) calculations reveal that the highest occupied molecular orbitals (HOMOs) of both molecules delocalize along the conjugated backbone, while the lowest unoccupied molecular orbitals (LUMOs) are primarily located on the electron acceptor, indicating the D–A feature of these two molecules. Compared to ODBP, OTBP boasts a longer conjugation length and more *π‐*electrons. Further DFT calculations indicate that OTBP shows a narrower bandgap (*E*_ge_ = 2.28 eV) and a larger transition dipole moment (*M*_ge_ = 3.03) compared to ODBP (*E*_ge_ = 2.54 eV, *M*_ge_ = 2.40). These molecular energy levels and electronic transition properties suggest that OTBP stands out as a promising candidate for BTHG imaging.^[^ ^48^, ^49^, ^50^ ^]^

Based on the theoretical prediction, we experimentally investigated the optical properties of OTBP and ODBP. As shown in Figure S3 (Supporting Information), both OTBP and ODBP exhibit typical AIE characteristics when they form aggregates in the DMSO/water mixture at high water volume fraction (*f*_w_). Notably, OTBP demonstrates a more obvious AIE effect than ODBP, indicating the stronger restriction of intramolecular motion (RIM) effect in OTBP aggregates. We then evaluated their solid‐state NLO efficiency using a multiphoton microscope (*λ*_ex_ = 1300 nm). Obviously, OTBP powders exhibit a stronger two‐photon fluorescence (2PF) and a higher BTHG efficiency compared to ODBP powders (Figure 2b). Similar results can be observed in their aggregates formed at *f*_w_ = 90% (Figure S4, Supporting Information), where stronger 2PF of OTBP aggregates results from its more robust RIM effect. To rationally explain the difference in the BTHG intensity, we evaluated the RE effects of OTBP and ODBP because matching the absorption band of the AIEgen to the virtual energy level of the BTHG process can enhance its efficiency.^[^ ^51^ ^]^ It was found that OTBP shows stronger absorption than ODBP at the wavelength range of 410–433 nm, indicating a stronger RE effect of THG in OTBP aggregates when the fundamental wavelength falls in a range from 1230 to 1300 nm (Figure 2c).

Inspired by the superior AIE and BTHG characteristics of OTBP aggregates, we further explored its size and NLO property changes during the aggregation process in the DMSO/water mixtures. As shown in Figure S5a (Supporting Information), OTBP demonstrates the largest particle size at *f*_w_ = 60%, suggestive of its highest aggregation degree. Further increasing *f*_w_, however, results in a dramatic decrease in particle size, consistent with the previously reported results.^[^ ^52^ ^]^ We then measured and compared the one‐photon fluorescence (1PF), 2PF, and BTHG of OTBP at different *f*_w_. OTBP generates the strongest 1PF and 2PF signal at *f*_w_ = 60%, while the BTHG signal remains strong at both *f*_w_ = 50% and 60%. Additionally, the 1PF, 2PF, and BTHG intensities of OTBP decrease with decreased particle size. These results indicate that the NLO effects of OTBP closely relate to the aggregation degree (Figure 2d; Figure S5b,c, Supporting Information). For both 1PF and 2PF, it is easy to understand that upon forming aggregates, the RIM effect can stabilize the excited state of OTBP, thus leading to the highest efficiency at *f*_w_ = 60%. However, as a unique process without electronic transitions, THG is still heavily influenced by the RIM effect, probably due to the increased *χ* ^(3)^ when OTBP molecules form condensed aggregates.^[^ ^47^ ^]^ Therefore, we speculate that the synergistic effect of RE and aggregation leads to a strong BTHG signal of OTBP in the aggregate state. Meanwhile, the above discussions highlight the importance of aggregation to both linear and non‐linear optics, though they are fundamentally different, reflecting the synergism in aggregate science.^[^ ^3^ ^]^ We also evaluate the BTHG wavelength under different excitation wavelengths, which offers flexibility in wavelength selection for diverse application environments. When the incident wavelength varied from 1230 to 1300 nm, the corresponding BTHG wavelength of OTBP showed a redshift accordingly (Figure 2e).

### 2.2 Aggregate‐State Mechanistic Studies for Enhanced BTHG Efficiency

**Figure 3:**
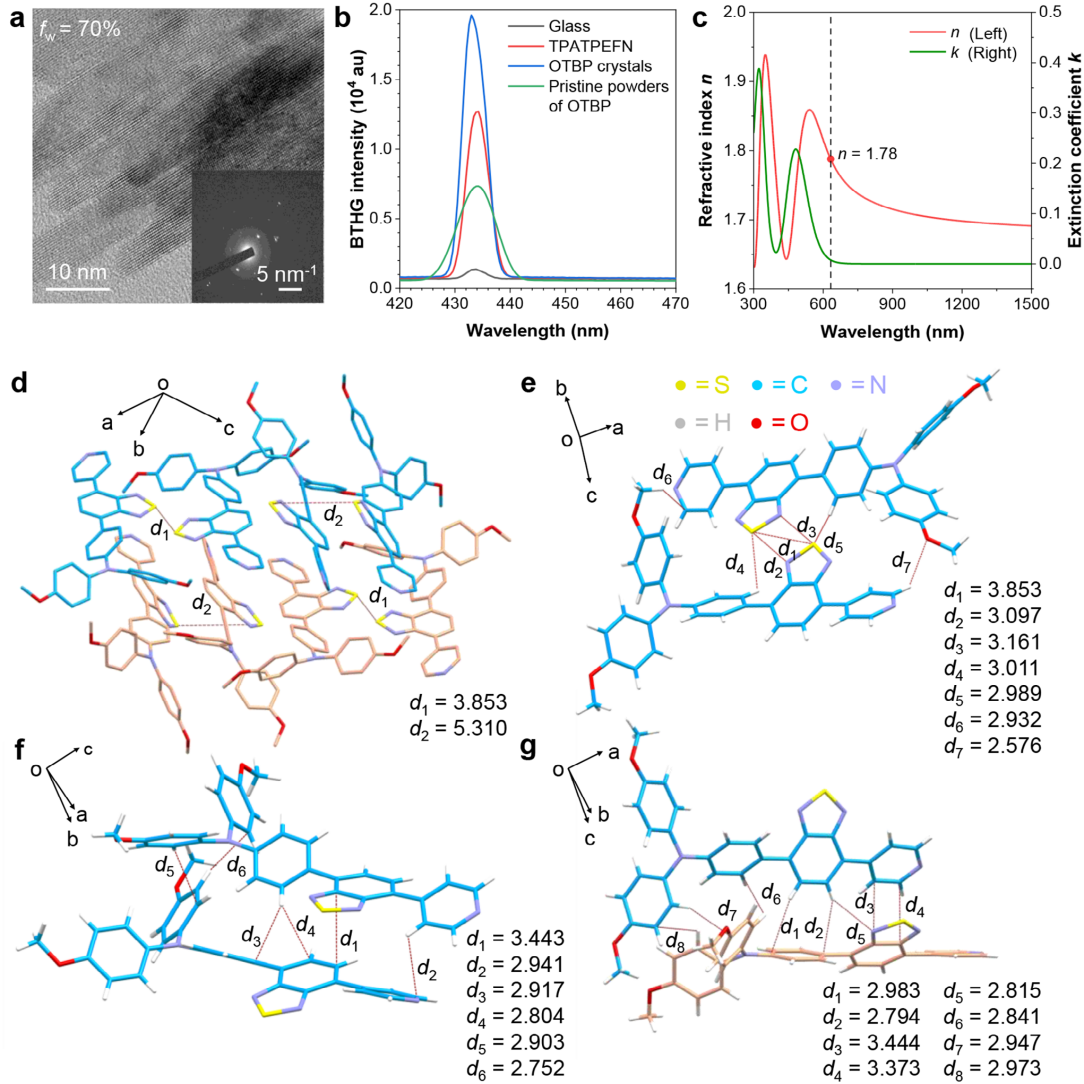
a) TEM image and selected area electron diffraction pattern of OTBP aggregates formed in a DMSO/water mixture at *f*_w_ = 70%. b) BTHG spectra of different BTHG contrast agents, including glass, TPATPEFN, OTBP crystals, and its pristine powders. *λ*_ex_ = 1300 nm; integration time = 0.099 s; Zoom = 100. c) Refractive index (*n*) and extinction coefficient (*k*) spectra of the spin‐coated thin film of OTBP. d–g) Molecular packing and intermolecular interaction analysis of single crystal of OTBP.

Our speculation toward the rigidification effect of OTBP can provide an explanation for enhanced BTHG efficiency from the molecular view. However, as many classic studies have pointed out, the THG process closely relates to the bulk property of materials. For example, the interface geometry could greatly improve the THG performance.^[^ ^16^, ^32^ ^]^ Because it is not a molecular‐level mechanism, we tried to further understand the THG enhancement mechanism of OTBP from the aggregate view. Here, we want to know whether the aggregation of OTBP molecules, if crystallization is possible to occur, can concurrently form an interface with a high refractive index for efficient THG. Transmission electron microscopy (TEM) was utilized to attain the microscopic structural details of OTBP aggregates. TEM images reveal that OTBP exhibits excellent crystallinity in various aggregation environments (**Figure** **3**a; Figure S6, Supporting Information). The neatly arranged lattice structures observed under TEM were confirmed by selected area electron diffraction. To prove the relationship between crystallinity and BTHG, we compared the BTHG intensities of various materials. As shown in Figure 3b, OTBP crystals demonstrate a much higher BTHG efficiency than pristine powders of OTBP under the excitation of 1300 nm. To our delight, these organic materials show much stronger BTHG than silicon dioxide glass, a classic inorganic THG material with *n* = 1.46,^[^ ^53^ ^]^ implying the advantage of *π*‐electron conjugation in boosting the THG signal of organic molecules. Additionally, we observed a stronger BTHG intensity from OTBP crystals than a previously reported top‐tier THG contrast agent called TPETPAFN,^[^ ^9^ ^]^ which further proves that OTBP can be a highly competitive BTHG bioimaging agent. We then prepared thin films of OTBP by spin‐coating to measure its refractive index. Polarized optical microscopy (POM) reveals that the obtained OTBP films form regular crystalline structures (Figure S7, Supporting Information), and they have a high refractive index of *n* = 1.78 at 633 nm measured by an ellipsometer (Figure 3c). These results indicate that OTBP can easily form crystals, ensuring the formation of a high refractive index interface for strong BTHG.

Single crystals of OTBP were grown via the diffusion method and then investigated by X‐ray diffractometer (Table S1, Supporting Information). The single crystal analysis reveals a staggered layer‐by‐layer arrangement (Figure 3d), where OTBP molecules within the same layer are closely connected, demonstrating excellent intermolecular interactions. Using the sulfur–sulfur distance between neighbouring benzothiadiazole units as a marker, two types of molecular arrangements of OTBP were identified within a layer (*d*_1_, *d*_2_ in Figure 3d). Interestingly, OTBP molecules in the interlayer also exhibit the same molecular arrangement pattern (Figure S8, Supporting Information), indicating that OTBP molecules are tightly interconnected in both directions and implying their superior crystallinity. Notably, OTBP molecules exhibit three distinct types of pairwise interactions within the crystal lattice. Figure 3e shows that a single OTBP molecule exhibits strong interactions with neighbouring benzothiadiazole (*d*_1_, *d*_2_, and *d*_3_), triphenylamine (*d*_4_ and *d*_5_), and pyridine moieties (*d*_6_ and *d*_7_). As shown in Figure 3f, there exist *π*–*π* interactions with a distance of 3.443 Å (*d*_1_), and C─H···*π* interactions with distances of 2.941 Å (*d*_2_), 2.917 Å (*d*_3_), 2.804 Å (*d*_4_), 2.903 Å (*d*_5_), and 2.752 Å (*d*_6_). Meanwhile, as shown in Figure 3g, multiple interactions exist in the interlayer, including T‐shaped C─H···*π* interactions between the benzothiadiazole and triphenylamine moieties with distances of 2.983 and 2.794 Å (*d*_1_ and *d*_2_), *π*–*π* interactions between the benzothiadiazole core and pyridine rings with distances of 3.444 and 3.373 Å (*d*_3_ and *d*_4_), C─H···N interactions between benzothiadiazole moieties with a distance of 2.815 Å (*d*_5_), and interactions between triphenylamine units with distances of 2.841, 2.947, and 2.973 Å (*d*_6_, *d*_7_, and *d*_8_). Therefore, the high crystallinity of OTBP originates from its distinct intermolecular interactions. With orderly molecular packing, OTBP molecules can form stable interfaces with a high refractive index, resulting in its exceptionally high BTHG efficiency.

### 2.3 Size Optimization for Enhanced BTHG Collection

**Figure 4:**
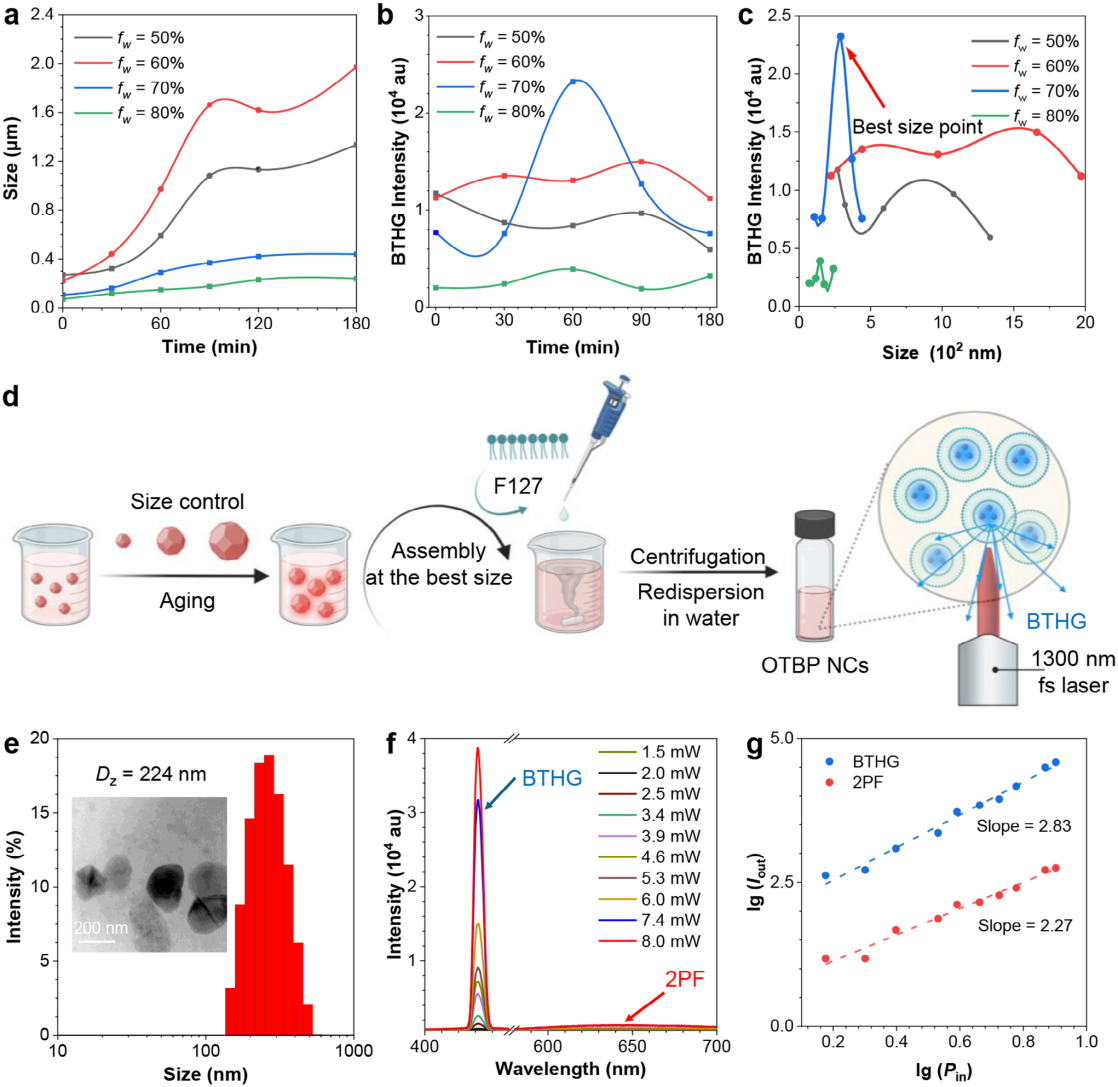
a) Size changes of OTBP NCs over time in DMSO/water mixtures with different *f*_w_. b) BTHG changes of OTBP over time in DMSO/water mixtures with different *f*_w_. c) Oscillating relationship between the BTHG intensity and the size of the NCs. d) Schematic illustration of size tuning strategy for the optimization of BTHG performance of OTBP NCs and their encapsulation with F‐127. e) Size distribution of the F‐127‐wrapped OTBP NCs. Inset: TEM image of the OTBP NCs. f) Luminescence spectra of the prepared OTBP NCs under different excitation powers. *λ*_ex_ = 1300 nm. g) Fitting lines of 2PF and BTHG intensities (*I*_out_) against excitation power (*P*_in_) of 1300‐nm laser. Both the horizontal and vertical axes are taken logarithms.

When conducting THG bioimaging in deep tissues or thick samples, collecting the BTHG signal is practically required. However, only 1% of the total THG signal can be directly generated in the backward direction due to momentum conservation.^[^ ^54^ ^]^ Therefore, optimizing the backward scattering of all THG signals through rational crystal engineering becomes crucial. Since the Mie scattering cross section closely relates to particle size,^[^ ^55^ ^]^ we tried to maximize the BTHG collection by tuning the size of OTBP NCs. By controlling the aggregation environment and aging time, the size of OTBP NCs can be finely tuned (**Figure** **4**a). In the DMSO/water mixtures, decreasing *f*_w_ can enlarge the size of NCs. By lengthening the aging time, the size of NCs can hold a general upward trend due to crystal growth upon dynamic dissolution and precipitation of OTBP molecules at the interface. At the same time, the BTHG signal was also found to fluctuate over aging time (Figure 4b; Figure S9, Supporting Information). These findings imply the underlying interplay between the NC size and the BTHG signal. By integrating the experimental data in Figure 4a,b, an optimal size point stands out among all the tested aggregation environments and aging times (Figure 4c). The oscillations of the backward scattering signal against the NC size agree well with the Mie scattering theory: scattering intensity oscillates with particle size, typically observed when *r*/*λ* > 0.1 (*r* is the radius of particle size and *λ* is the emission wavelength of the THG).^[^ ^55^, ^56^, ^57^, ^58^ ^]^ At the best size point, where the strongest BTHG is observed in Figure 4c, the radius *r* of OTBP NCs is 124 nm, and thus *r*/*λ* is calculated to be ≈ 0.28, representing a typical Mie scattering feature. This result also indicates the effectiveness of size optimization of OTBP NCs for the BTHG enhancement.

To clarify that the enhanced BTHG signal does result from the size‐wavelength matching rather than the crystallinity variation caused by aging process, the crystallographic properties of OTBP NCs with or without the treatment of aging were studied by powder X‐ray diffraction (PXRD). The results show that OTBP NCs show similar and obvious diffraction patterns even after 1 h‐aging. Therefore, it is concluded that the aging process only induces size change without changing the crystallinity of OTBP NCs (Figure S10, Supporting Information). In addition, POM images support the same conclusion (Figure S11, Supporting Information). Of note, the pristine powders of OTBP can only reveal faint PXRD peaks, proving that the high crystallinity of OTBP NCs is successfully induced in the DMSO/water mixture (*f*_w_ = 70%). In addition to the backward‐emitted THG, the backward‐scattered FTHG signal also contributes to the BTHG signal. Accordingly, we further acquired the BTHG and FTHG images of OTBP NCs during aging process at *f*_w_ = 70% and analyzed their mean intensity via Image J (Figure S12, Supporting Information). The BTHG change maintains a trend similar to the data presented in Figure 4b, while the FTHG signal constantly boosts over aging time, which is attributed to the increased size and concentration of OTBP NCs (Figure S13, Supporting Information). Hence, the above results clearly expound the significant contribution of backward‐scattered FTHG to the total backward‐collected THG signal.

After optimizing the size of OTBP NCs and ensuring their crystallinity, we encapsulated the NCs prepared at the best size point with a widely used surfactant namely F‐127 to yield water‐soluble NCs for bioimaging applications (Figure 4d).^[^ ^59^, ^60^, ^61^ ^]^ As confirmed by dynamic light scattering (Figure 4e), the F‐127‐wrapped NCs reveal an average particle size of 224 nm. Their morphology and size distribution were further validated by TEM. According to reports, glomerular capillaries show pores ranging from 60 to 80 nm in diameter, while capillaries in the liver and spleen can feature fenestrations up to 150 nm.^[^ ^62^ ^]^ Therefore, from a perspective of in vivo transport of nanoparticles, this optimal particle size is also conducive to prolonging the systemic circulation time of the F‐127‐wrapped NCs. The BTHG performance of the water‐soluble NCs was investigated upon 1300‐nm laser irradiation. As shown in Figure 4f, a significant BTHG signal was generated at a very low excitation power of 2.5 mW (pulse energy of ≈30 pJ). The intensity of the THG spectra is proportional to the cube of the incident laser power (Figure 4g), proving that the 433 nm emission is due to the THG process rather than other luminescent phenomena. We can also observe clear but much weaker 640 nm 2PF signals, which are proved by the square relationship with the incident intensity. These results demonstrate that the BTHG signal outperforms the 2PF signal when the NCs are irradiated by 1300 nm laser, and the encapsulation of the NCs using F‐127 does not affect the BTHG performance.

### 2.4 In Vivo Bio–Application

**Figure 5:**
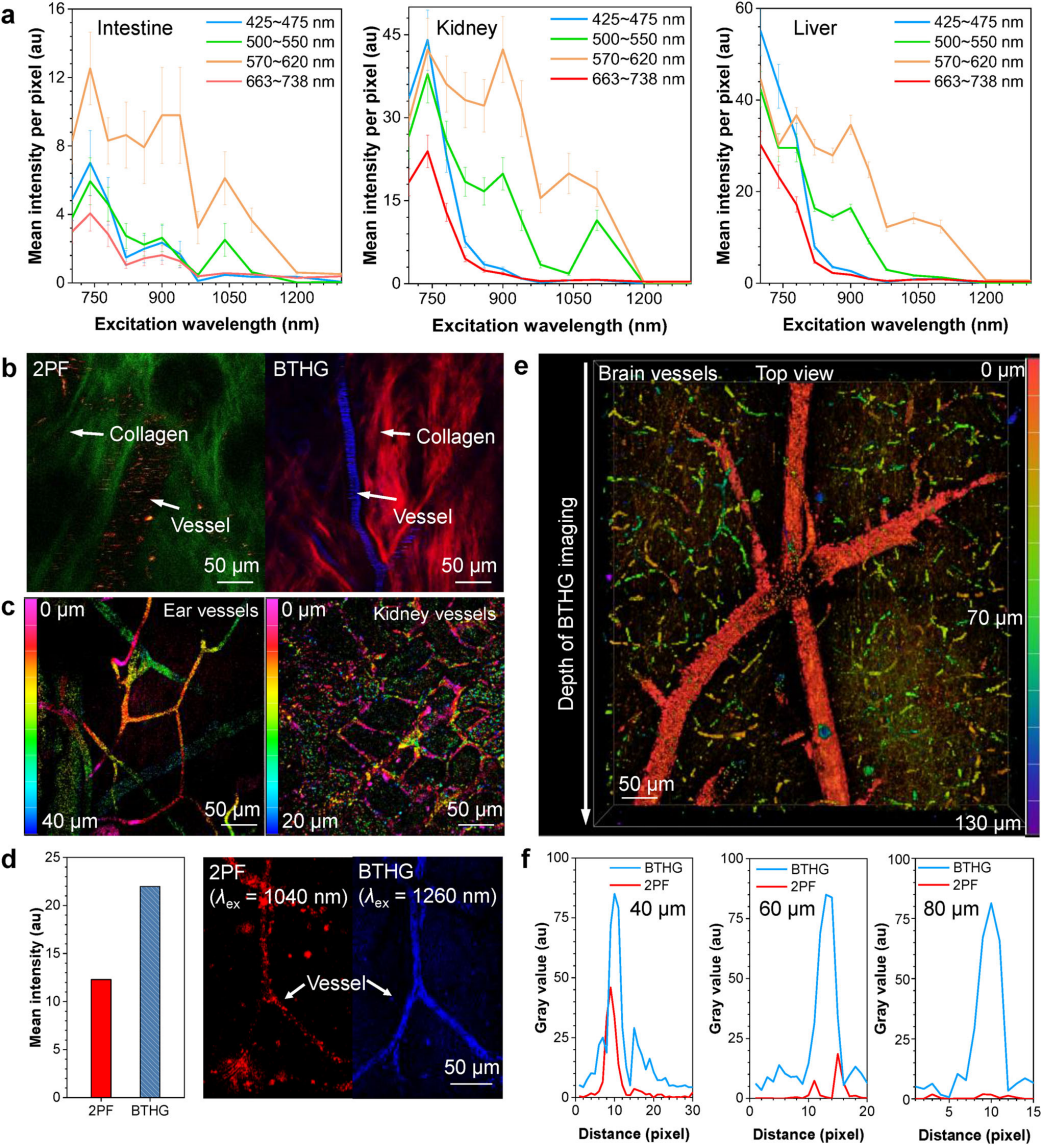
a) Autofluorescence intensity changes at various excitation wavelengths in different tissues. *λ*_ex_ = 700–1300 nm; *λ*_em_ = 425–475 nm, 500–550 nm, 570–620 nm, 663–738 nm. b) 2PF and BTHG imaging of mouse ear at a post‐objective laser power of 8 mW after intravenous injection of OTBP NCs. The green signal obtained upon 1040‐nm excitation or the red signal obtained upon 1300‐nm excitation is the second‐harmonic generation from collagen fibers; *λ*_ex_ = 1040 nm for 2PF; *λ*_ex_ = 1300 nm for BTHG. c) 3D Z‐depth coding reconstruction of 40‐µm‐thick mouse ear and 20‐µm‐thick kidney vessels enabled by BTHG imaging. d) 2PF and BTHG imaging of brain vessels using OTBP NCs at a post‐objective laser power of 15 mW. The mean intensity of 2PF and BTHG are also shown; *λ*_ex_ = 1040 nm for 2PF; *λ*_ex_ = 1260 nm for BTHG. e) BTHG imaging of brain vessels at different depths. *λ*_ex_ = 1260 nm. f) 2PF and BTHG signal profiles along the dashed line across the brain vessel at different depths. The dashed lines are shown in Figure S15 (Supporting Information).

When conducting multiphoton in vivo imaging, photodamage remains a critical issue. If incident optical power exceeds 20 mW, photodamage increases linearly with laser power.^[^ ^29^ ^]^ To circumvent this issue, we employed an ultra‐low 8‐mW (100‐pJ) excitation laser power (pulse energy) at *λ*_ex_ = 1200–1300 nm for non‐invasive THG angiography in vivo. At this wavelength range, laser excitation did not induce noticeable background interference at THG detection band (425–475 nm, blue curves in **Figure** **5**a) in various tissues (Figures S14–S16, Supporting Information). By contrast, obvious autofluorescence of tissues appeared at *λ*_ex_ = 700–1200 nm. These results confirm that the NIR‐II excitation window of 1200–1300 nm is advantageous to high signal‐to‐background ratio (SBR) BTHG imaging.^[^ ^22^ ^]^

Leveraging the exceptional BTHG efficiency of water‐soluble OTBP NCs, we successfully captured in vivo images of murine ear vasculature upon 1300 nm laser excitation at low excitation power (Figure 5b). Concurrently, OTBP NCs render a typical AIE signature of large Stokes shift with an emission wavelength of 640 nm, which enables the separation of BTHG from 2PF, thereby allowing for dual verification of signal information while providing opportunities for multimodal imaging. Apparently, the BTHG signal provided by OTBP NCs exhibited more superior contrast compared to their 2PF signal under 1040 nm excitation at an equivalent post‐objective laser power of 8 mW. This high contrast further benefits the exploration of OTBP NCs for 3D vascular reconstruction in tissues. Consequently, 3D Z‐depth coding images of 38‐um‐thick murine ear vasculature and 18 µm‐thick renal cortex vasculatures were finely reconstructed with low background noise (Figure 5c), suggesting that OTBP NCs can serve as a versatile contrast agent for angiography. To verify the deep tissue imaging capability of OTBP NCs, we conducted low‐power BTHG cerebral angiography. Even at a post‐objective laser power of 15 mW, the imaging contrast of BTHG still outperforms that of 2PF (Figure 5d). More importantly, under such low power, the imaging depth of OTBP NCs can still reach up to 130 µm (Figure 5e), demonstrating their excellent capability in BTHG imaging. In comparison, a 100 mW post‐objective power is typically required for deep brain imaging.^[^ ^63^ ^]^ Moreover, the image clarity and intensity of 2PF rapidly diminished at such a low excitation power, while the BTHG imaging could visualize small vessels at 80 µm depth (Figure 5f; Figure S17, Supporting Information).

**Figure 6:**
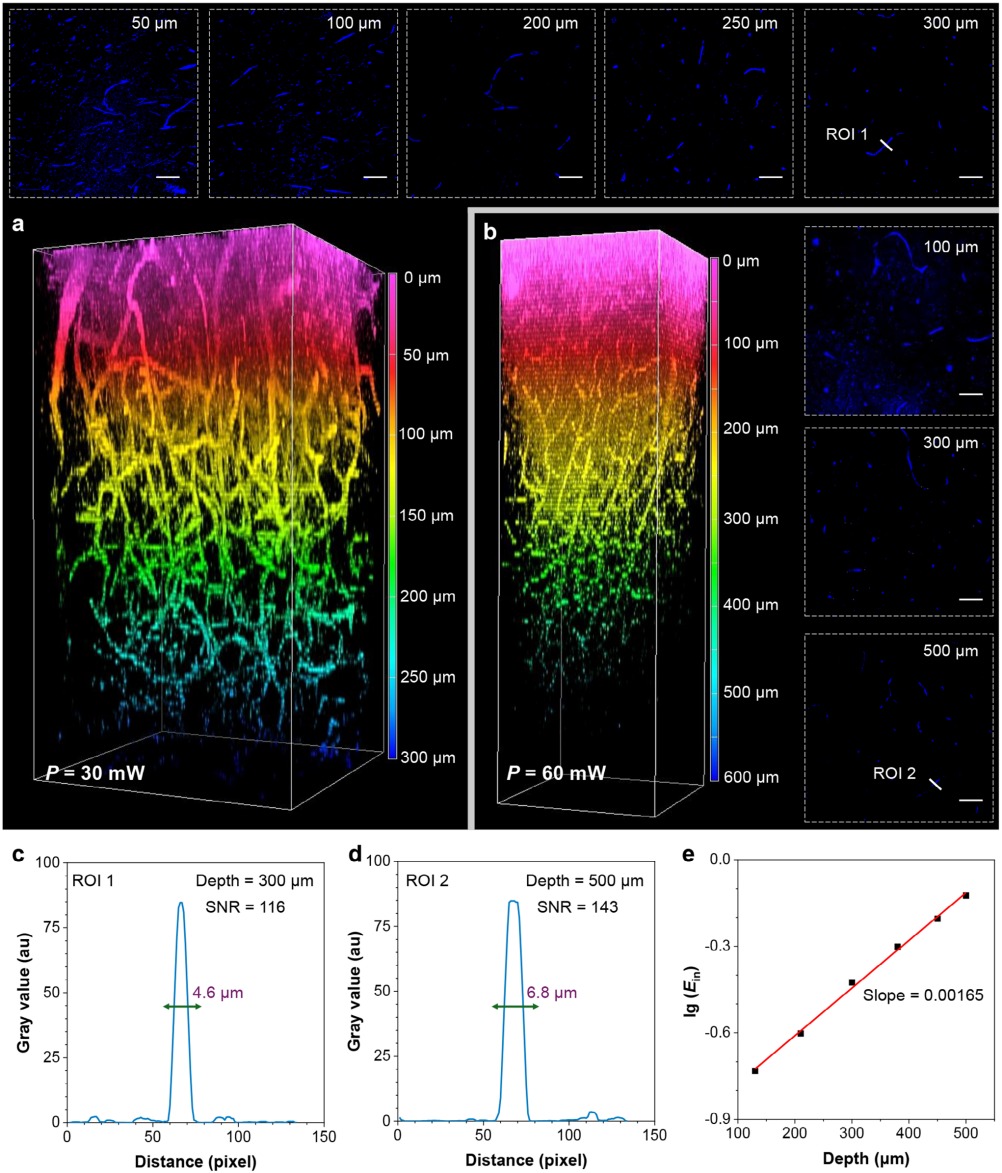
a,b) 3D reconstructions of brain vessels at different laser powers, together with BTHG images at various depths. *λ*_ex_ = 1260 nm. c,d) BTHG signal profiles along the white line across the brain vessel at different depths (ROI 1 and RO2, as indicated in Figure 6a,b). e) Fitting line of BTHG imaging depth against the logarithm of excitation pulse energy (*E*_in_) of 1260‐nm laser.

With increased pulse energy, we then explore the potential of OTBP NCs in deep‐brain vascular imaging. As the pulse energy is increased to 30 mW (≈375 pJ), the imaging depth can reach 300 µm (**Figure** **6**a). If the energy doubles to 60 mW (≈750 pJ), brain vessels can still be observed at 500 µm (Figure 6b). For a more comprehensive assessment of image quality, we constructed vascular transects at different depths and plotted pixel intensity profiles for white lines across tiny capillaries (Figure 6c,d). By virtue of the high BTHG efficiency of OTBP NCs, we can clearly visualize 4.6‐µm microvessel at a 300 µm depth and 6.8‐µm microvessel at a 500 µm depth with high SNRs of 114 and 143, respectively, indicative of strong vascular confinement. Notably, our BTHG imaging performance surpasses previous BTHG studies, where using a similar low‐power laser only achieved superficial skin imaging.^[^ ^64^ ^]^ Besides, leveraging the 1300‐nm excitation can effectively mitigate water absorption to result in less energy attenuation.^[^ ^65^ ^]^ Hence, the optimized third‐order nonlinear excitation window, coupled with the high efficiency of the prepared BTHG contrast agent, enables us to achieve superior image quality while under low pulse energies. When applying diverse pulse energies to conduct cerebral angiography with the NCs, we observed a linear relationship between the imaging depth and the logarithm of pulse energy, which is consistent with previous reports (Figure 6e).^[^ ^22^ ^]^ Supposing that the NCs receive sufficient pulse energy, they have the potential to achieve deeper imaging depths. Based on our experimental data, an imaging depth of 1.35 mm can be actualized when the pulse energy is increased to 20 nJ, which is typically used for deep‐brain imaging.^[^ ^66^, ^67^ ^]^ These calculations are elaborated in the Supporting Information. At last, the biocompatibility of OTBP NCs was assessed in mice by intravenous administration with OTBP NCs once a day for one week. Histological examination by H&E staining revealed no discernible organ damages or inflammatory responses. The mice maintained normal organ morphology (Figure S18, Supporting Information), affirming the good biocompatibility of OTBP NCs.

## 3 Conclusion

In conclusion, we have developed an ultra‐efficient BTHG contrast agent called OTBP synthesized by linking an electron‐rich 4,4′‐dimethoxytriphenylamine donor to electron‐deficient benzothiadiazole and pyridine moieties. Compared to ODBP, OTBP demonstrates better *π*‐electron conjugation and a larger transition dipole moment, which benefit the BTHG enhancement. In the excitation window of 1230–1300 nm, the absorption band of OTBP is in proximity to its RE region, thus contributing to the BTHG improvement. In addition to the BTHG enhancement at the molecular level, the bulk properties of OTBP have also been investigated in detail. Thanks to the high crystallization tendency of OTBP, the NCs can be easily prepared upon aggregation in the DMSO/water mixture, thereby providing a high refractive index interface for further BTHG enhancement. Altering the aggregation environment and aging time can lead to adjustable size and BTHG intensity of OTPB NCs. A best size point that perfectly matches the incident wavelength has been successfully identified among the oscillating curves, where the Mie scattering cross‐section is maximized to achieve optimal BTHG collection efficiency. Based on the OTBP NCs with the highest BTHG efficiency, we fabricated water‐soluble BTHG contrast agents using F‐127 as a surfactant. This enables 3D reconstruction of tissue vasculature with high SBR and biocompatibility at extremely low 100‐pJ pulse energy (8‐mW excitation power), thus dramatically mitigating the risk of photodamage. At a high laser power of 60 mW, the BTHG signal of OTBP NCs enables the visualization of microvessels at 500 µm with a high SNR of 143. By taking the NLO enhancement effect at both the molecular and aggregate levels into consideration, we rationally construct a promising BTHG contrast agent for low‐power THG angiography in vivo. This methodology is expandable to the development of other functional aggregates, ultimately contributing to the advancement of aggregate science.

## 4 Experimental Section

### Materials

All the chemicals and reagents were commercially available, and the solvents for chemical reactions were distilled before use. All air and moisture‐sensitive reactions were carried out in flame‐dried glassware under a nitrogen atmosphere. Dulbecco's modified eagle medium (DMEM) cell culture and fetal bovine serum (FBS) was purchased from Gibco. Trypsin (0.25%) and penicillin‐streptomycin solution (100×) were purchased from MesGen Biotechnology.

### Instruments

^1^H and ^13^C NMR spectra were measured on a Bruker AVIII 400 NMR spectrometer. High‐resolution mass spectra (HRMS) were recorded on a Water's Xevo G2‐XS Tof mass spectrometer with an Electrospray source. UV–vis–NIR absorption spectra were measured using a Shimadzu UV–vis Spectrophotometer UV‐2600i. PL spectra were recorded on a Horiba Fluorolog‐3 Spectrofluorometer. The dynamic light scattering (DLS) data were recorded by Malvern Zetasizer Pro. Transmission Electron Microscope (TEM) images were recorded on a JEM 2100F microscope. Two‐photon and BTHG images were taken by using a wavelength‐tunable (700–1300 nm) near‐infrared femtosecond laser (InSight X3, Spectra‐Physics) as the light source, and each image was captured using a Nikon Eclipse Inverted Multiphoton Microscope (A1MP + Eclipse Ti‐2E, Nikon instrument Inc., Japan) with a water‐immersed 40× (NA = 1.15) objective. Multiphoton spectra were collected by a CCD‐cooled spectrometer (iDus 401 plus shamrock 193i, ANDOR, Oxford Instruments) attached on the backside port of the multiphoton microscope. Ellipsometric measurement was performed by RC2 UI (J. A. Woollam Co., Inc). Powder X‐Ray Diffractometer (PXRD) patterns were carried out in the reflection mode at room temperature using a 2.2 kW Empyrean X‐ray Diffraction System (PANalytical, Netherland). 3D organ slicing was processed by a vibratome (VT1200S, Leica Biosystems Nussloch GmbH). Polarized optical microscope (POM) images were taken through Olympus BH‐2. Particles concentration was processed by Particle Metrix ZetaView NTA.

### Crystal Growing

OTBP powders were completely dissolved in a glass bottle vial using DCM. The obtained solution was placed in a hexane environment outside. The slow evaporation of hexane into the DCM solution resulted in the formation of OTBP crystals, which were further collected for X‐ray crystallographic analysis. CCDC 2369657 (OTBP) contains the supplementary crystallographic data for this paper. These data can be obtained free of charge from the Cambridge Crystallographic Data Centre via http://www.ccdc.cam.ac.UK/data_request/cif .

### Preparation of Crystalline Thin Films of OTBP and Ellipsometry Measurement

OTBP powders were completely dissolved in a dichlorobenzene/DMAC mixture at a concentration of 25 mg mL^−1^ (1:1, v/v). Large particles in the solution were filtered out using a 0.4 µm membrane filter. Subsequently, spin‐coating was done by slowly adding 40 uL of the filtered solution on a silicon wafer at a spin speed of 500 rpm and the spin‐coating time was 40 s. The wafer was dried at 80 °C. Eventually, the dried silicon wafer was tested on an ellipsometer with an incident angle of 75° and a fitting refractive index range of 300–1700 nm.

### Animal Model

Balb/C mice aged 6–8 weeks were used for the experiments and all procedures were approved by the Animal Research Core of the Faculty of Health Sciences, University of Macau. All animals are used and maintained in accordance with the NIH Guide for the Care and Use of Laboratory Animals, PHS Animal Welfare Policy, and Animal Welfare (Ethics number: UMARE‐063‐2023). Before imaging experiments, all mice were anesthetized through the abdominal injection of avertin. Three mice were independently investigated for each group of imaging experiments.

### Autofluorescence Imaging of Organs

The organs were harvested from the mouse model and stored in the cold PBS. Afterward, they were cut into 200‐µm‐thick slices with a vibratome within 1 h. For the detection of autofluorescence, the laser power was fixed at 8 mW (100 pJ) and then captured fluorescent images under an excitation wavelength of 700–1300 nm.

### In Vivo Imaging of Mouse Ear Vessels

F‐127‐wrapped OTBP NCs (1 mm) in phosphate‐buffered saline (PBS) were prepared. 100 µL of the NC solution was injected into the mice through the tail vein. After 30 s of injection, the BTHG and second harmonic generation (SHG) signals were epi‐collected by the same objective, reflected by a multiphoton dichroic beam splitter, and detected by four photomultiplier tubes (PMTs). In the BTHG and two‐photon (2PF) imaging experiments, the laser power was equalized to 8 mW (100 pJ).

### 3D Reconstruction of Renal Cortex and Brain Vessels

After OTBP NCs were intravenously injected into the mouse model, organs were harvested from the mouse model and stored in the cold PBS. Afterward, they were cut into 800‐µm‐thick slices with a vibratome within 1 h. The excitation wavelength and laser power were selected for individual 3D reconstruction (*P_in_* = 8 mW, *E*_in_ = 100 pJ and *λ*_ex_ = 1300 nm for renal cortex vessel imaging; *P*_in_ = 15, 30, or 60 mW, *E*_in_ = ≈200, 375, or 750 pJ and *λ*_ex_ = 1260 nm for brain vessel imaging). The flat was a 2 × 2 large image under a 40 × objective (Image pixels = 512 × 512 and dwell time = 1.1 s).

## Conflict of Interest

The authors declare no conflict of interest.

## Supporting information



Supporting Information

## Data Availability

The data that support the findings of this study are available from the corresponding author upon reasonable request.

## References

[1] B. Z. Tang , Aggregate 2020, 1, 4.

[2] B. Liu , B. Z. Tang , Angew. Chem., Int. Ed. Engl. 2020, 59, 9788. 32449572 10.1002/anie.202005345

[3] Y. Tu , Z. Zhao , J. W. Lam , B. Z. Tang , Matter 2021, 4, 338.

[4] H. Wang , Q. Li , P. Alam , H. Bai , V. Bhalla , M. R. Bryce , M. Cao , C. Chen , S. Chen , X. Chen , ACS Nano. 2023, 17, 14347. 37486125 10.1021/acsnano.3c03925 PMC10416578

[5] W. He , R. T. K. Kwok , Z. Qiu , Z. Zhao , B. Z. Tang , J. Am. Chem. Soc. 2024, 146, 5030. 38359354 10.1021/jacs.3c09892

[6] C. Xu , H. Shen , T.‐M. Liu , R. T. Kwok , J. W. Lam , B. Z. Tang , iScience 2023, 26, 106568. 37128609 10.1016/j.isci.2023.106568 PMC10148129

[7] R. Xu , P. Zhang , Q. Shen , Y. Zhou , Z. Wang , Y. Xu , L. Meng , D. Dang , B. Z. Tang , Coord. Chem. Rev. 2023, 477, 214944.

[8] Z. Zheng , D. Li , Z. Liu , H. Q. Peng , H. H. Sung , R. T. Kwok , I. D. Williams , J. W. Lam , J. Qian , B. Z. Tang , Adv. Mater. 2019, 31, 1904799. 10.1002/adma.201904799 31523871

[9] J. Qian , Z. Zhu , A. Qin , W. Qin , L. Chu , F. Cai , H. Zhang , Q. Wu , R. Hu , B. Z. Tang , Adv. Mater. 2015, 27, 2332. 25711371 10.1002/adma.201500141

[10] X. Han , F. Ge , J. Xu , X. H. Bu , Aggregate 2021, 2, 28.

[11] A. R. Suthya , C. H. Wong , J. H. Bourne , Front. Immunol. 2024, 15, 1372996. 38817606 10.3389/fimmu.2024.1372996 PMC11137164

[12] C.‐C. Zhang , J.‐Y. Zhang , J.‐R. Feng , S.‐T. Liu , S.‐J. Ding , L. Ma , Q.‐Q. Wang , Nanoscale 2024, 16, 5960. 38446099 10.1039/d3nr06675d

[13] Y. Wang , F. Heymann , M. Peiseler , Gut 2024, 73, 331739. 10.1136/gutjnl-2023-331739 38777574

[14] B. Weigelin , G.‐J. Bakker , P. Friedl , IntraVital 2012, 1, 32. 29607252 10.4161/intv.21223 PMC5858865

[15] C.‐F. Chang , C.‐Y. Chen , F.‐H. Chang , S.‐P. Tai , C.‐Y. Chen , C.‐H. Yu , Y.‐B. Tseng , T.‐H. Tsai , I.‐S. Liu , W.‐F. Su , Opt. Express. 2008, 16, 9534. 18575520 10.1364/oe.16.009534

[16] S. Witte , A. Negrean , J. C. Lodder , C. P. De Kock , G. T. Silva , H. D. Mansvelder , M. L. Groot , Proc. Natl. Acad. Sci. 2011, 108, 5970. 21444784 10.1073/pnas.1018743108 PMC3076839

[17] G. Raju , S. Nayak , N. Acharya , M. Sunder , Y. Kistenev , N. Mazumder , J. Biophotonics. 2024, 17, 202300360. 10.1002/jbio.202300360 38168892

[18] S. Zhang , L. Liu , S. Ren , Z. Li , Y. Zhao , Z. Yang , R. Hu , J. Qu , Opto‐Electron. Adv. 2020, 3, 200003.

[19] X. Wang , D. Zhang , X. Zhang , Y. Xing , J. Wu , X. Sui , X. Huang , G. Chang , L. Li , Technol. Cancer Res. Treat. 2022, 21, 15330338221133244. 36379591 10.1177/15330338221133244 PMC9676310

[20] C.‐K. Chen , T.‐M. Liu , Biomed. Opt. Express. 2012, 3, 2860. 23162724 10.1364/BOE.3.002860 PMC3493243

[21] C.‐K. Tsai , T.‐D. Wang , J.‐W. Lin , R.‐B. Hsu , L.‐Z. Guo , S.‐T. Chen , T.‐M. Liu , Biomed. Opt. Express. 2012, 4, 178. 23304657 10.1364/BOE.4.000178 PMC3539194

[22] T. Wang , C. Xu , Optica 2020, 7, 947.

[23] W. Choi , B. Park , S. Choi , D. Oh , J. Kim , C. Kim , Chem. Rev. 2023, 123, 7379. 36642892 10.1021/acs.chemrev.2c00627

[24] C. W. Lee , P. C. Wu , I. L. Hsu , T. M. Liu , W. H. Chong , C. H. Wu , T. Y. Hsieh , L. Z. Guo , Y. Tsao , P. T. Wu , Small 2019, 15, 1805086. 10.1002/smll.201805086 30925031

[25] C.‐F. Chang , H.‐C. Chen , M.‐J. Chen , W.‐R. Liu , W.‐F. Hsieh , C.‐H. Hsu , C.‐Y. Chen , F.‐H. Chang , C.‐H. Yu , C.‐K. Sun , Opt. Express. 2010, 18, 7397. 20389762 10.1364/OE.18.007397

[26] W. Wu , S. Tang , IEEE J. Sel. Top. Quantum Electron. 2023, 29, 700309.

[27] E. Gavgiotaki , G. Filippidis , V. Tsafas , S. Bovasianos , G. Kenanakis , V. Georgoulias , M. Tzardi , S. Agelaki , I. Athanassakis , Sci. Rep. 2020, 10, 11055. 32632110 10.1038/s41598-020-67857-y PMC7338369

[28] V. Shcheslavskiy , G. Petrov , V. V. Yakovlev , Appl. Phys. Lett. 2003, 82, 3982.

[29] R. Galli , O. Uckermann , E. F. Andresen , K. D. Geiger , E. Koch , G. Schackert , G. Steiner , M. Kirsch , PLoS One 2014, 9, 110295. 10.1371/journal.pone.0110295 PMC4208781 25343251

[30] N. Ji , J. C. Magee , E. Betzig , Nat. Methods. 2008, 5, 197. 18204458 10.1038/nmeth.1175

[31] H. Ticha , L. Tichy , J. Optoelectron , Adv. Mater. 2002, 4, 381.

[32] T. Y. Tsang , Phys. Rev. A 1995, 52, 4116. 9912728 10.1103/physreva.52.4116

[33] P. F. Bordui , M. M. Fejer , Annu. Rev. Mater. Sci. 1993, 23, 321.

[34] M. Liu , H. S. Quah , S. Wen , Z. Yu , J. J. Vittal , W. Ji , Chem. Mater. 2016, 28, 3385.

[35] A. Dilli Rani , M. Nageshwari , C. Rathika Thaya Kumari , P. Ramesh , P. Sangeetha , G. Vinitha , M. Lydia Caroline , S. Kumaresan , J. Mater. Sci. Mater. Electron. 2023, 34, 1476.

[36] V. L. Roggli , J. Mastin , J. D. Shelburne , M. Roe , A. R. Brody , *Inflammatory Cells Lung Dis*., CRC Press, ISBN:9780429274435 2023.

[37] R. Mohammapdour , H. Ghandehari , Adv. Drug Delivery Rev. 2022, 180, 114022. 10.1016/j.addr.2021.114022 PMC8898339 34740764

[38] S. L. Palencia , A. M. Buelvas , M. S. Palencia , Curr. Chem. Biol. 2015, 9, 10.

[39] S. Helmig , D. Walter , J. Putzier , H. Maxeiner , S. Wenzel , J. Schneider , Mol. Med. Rep. 2018, 17, 8518. 29693699 10.3892/mmr.2018.8923

[40] M. Sheik‐Bahae , M. P. Hasselbeck , Handb. Opt. 2000, 4, 16.

[41] B. Shen , L. Liu , Y. Li , S. Ren , J. Yan , R. Hu , J. Qu , Adv. Opt. Mater. 2020, 8, 1901981.

[42] I. Alonso Calafell , L. A. Rozema , A. Trenti , J. Bohn , E. J. Dias , P. K. Jenke , K. S. Menghrajani , D. Alcaraz Iranzo , F. J. García de Abajo , F. H. Koppens , Adv. Opt. Mater. 2022, 10, 2200715.

[43] J. Ferrer Ortas , P. Mahou , S. Escot , C. Stringari , N. B. David , L. Bally‐Cuif , N. Dray , M. Négrerie , W. Supatto , E. Beaurepaire , Light: Sci. Appl. 2023, 12, 29. 36702815 10.1038/s41377-022-01064-4 PMC9879988

[44] S. Yang , P. A. Yin , L. Li , Q. Peng , X. Gu , G. Gao , J. You , B. Z. Tang , Angew. Chem., Int. Ed. 2020, 59, 10136. 10.1002/anie.201914437 31872942

[45] B. Z. Tang , B. Liu , Chem 2020, 6, 2770.

[46] J. Zhang , B. He , W. Wu , P. Alam , H. Zhang , J. Gong , F. Song , Z. Wang , H. H. Sung , I. D. Williams , J. Am. Chem. Soc. 2020, 142, 14608. 32787264 10.1021/jacs.0c06305

[47] M. O. Senge , M. Fazekas , E. G. Notaras , W. J. Blau , M. Zawadzka , O. B. Locos , E. M. Ni Mhuircheartaigh , Adv. Mater. 2007, 19, 2737.

[48] C. B. Gorman , S. R. Marder , Proc. Natl. Acad. Sci. 1993, 90, 11297. 11607441 10.1073/pnas.90.23.11297 PMC47969

[49] S. R. Marder , MRS Bull. 2016, 41, 53.

[50] F. Meyers , S. R. Marder , B. M. Pierce , J.‐L. Bredas , J. Am. Chem. Soc. 1994, 116, 10703.

[51] C.‐H. Yu , S.‐P. Tai , C.‐T. Kung , W.‐J. Lee , Y.‐F. Chan , H.‐L. Liu , J.‐Y. Lyu , C.‐K. Sun , Opt. Lett. 2008, 33, 387. 18278119 10.1364/ol.33.000387

[52] S. Gan , W. Wu , G. Feng , Z. Wang , B. Liu , B. Z. Tang , Small 2022, 18, 2202242. 10.1002/smll.202202242 35652497

[53] W. Pliskin , R. Esch , J. Appl. Phys. 1965, 36, 2011.

[54] D. S. James , P. J. Campagnola , BME Front. 2021, 2021, 24. 10.34133/2021/3973857 PMC10521653 37849910

[55] K. Maciej , Comp. Meth. Sci. Tech. 2010, 2 , 107.

[56] C. F. Bohren , D. R. Huffman , Absorption and Scattering of Light by Small Particles, John Wiley & Sons, Hoboken, New Jersey 2008.

[57] K. Nassau , The Physics and Chemistry of Color: the Fifteen Causes of Color, Wiley‐VCH, Weinheim 2001.

[58] S. Gibilisco , Optics demystified., McGraw Hill Professional, ISBN:007178215X 2009.

[59] C. Xu , X. Ou , B. Wang , H. Shen , J. Liu , X. Yang , Q. Zhou , J. H. Chau , H. H. Sung , G. Xing , J. Am. Chem. Soc. 2024, 146, 4851. 38346857 10.1021/jacs.3c13252

[60] R. Zhang , P. Shen , Y. Xiong , T. Wu , G. Wang , Y. Wang , L. Zhang , H. Yang , W. He , J. Du , Natl. Sci. Rev. 2024, 11, nwad286. 38213521 10.1093/nsr/nwad286 PMC10776353

[61] B. Yu , M. Liu , L. Jiang , C. Xu , H. Hu , T. Huang , D. Xu , N. Wang , Q. Li , B. Z. Tang , Adv. Healthcare Mater. 2024, 13, 2303643. 10.1002/adhm.202303643 38115727

[62] M. K. Danquah , X. A. Zhang , R. I. Mahato , Adv. Drug Delivery Rev. 2011, 63, 623. 10.1016/j.addr.2010.11.005 21144874

[63] B. Weigelin , G.‐J. Bakker , P. Friedl , J. Cell Sci. 2016, 129, 245. 26743082 10.1242/jcs.152272

[64] Y.‐H. Liao , Y.‐H. Su , Y.‐T. Shih , W.‐S. Chen , S.‐H. Jee , C.‐K. Sun , J. Biomed. Opt. 2020, 25, 014504. 10.1117/1.JBO.25.1.014504 PMC7008507 31777224

[65] M. Wang , C. Wu , D. Sinefeld , B. Li , F. Xia , C. Xu , Biomed. Opt. Express. 2018, 9, 3534. 30338138 10.1364/BOE.9.003534 PMC6191617

[66] N. G. Horton , K. Wang , D. Kobat , C. G. Clark , F. W. Wise , C. B. Schaffer , C. Xu , Nat. Photonics. 2013, 7, 205. 24353743 10.1038/nphoton.2012.336 PMC3864872

[67] A. Lanin , M. Pochechuev , A. Chebotarev , I. Kelmanson , D. Bilan , D. Kotova , V. Tarabykin , A. Ivanov , A. Fedotov , V. Belousov , Opt. Lett. 2020, 45, 836. 32058483 10.1364/OL.45.000836

