# Supplementary material for: From Molecule to Aggregate: Designing AIE Nanocrystals for Low‐Power Backward Third‐Harmonic Generation Angiography
Source: Adv Mater. 2025 Apr 1;37(20):2414419. doi: 10.1002/adma.202414419 (PMC12087731; doi:10.1002/adma.202414419)
Supplement: Supplementary file 1 — Supporting Information [file ADMA-37-2414419-s001.docx]

Supporting Information

**From Molecule to Aggregate: Designing AIE Nanocrystals for Low-Power Backward Third-Harmonic Generation Angiography**

*Lidong Du, Hanchen Shen, Changhuo Xu^*^, Xinyan Zhu, Bingnan Wang, Qingqing Zhou, Chunxi Liu,* *Herman H. Y. Sung, Ryan T. K. Kwok, Jacky W. Y. Lam^*^, Quan Zhou, Tzu-Ming Liu^*^, Ben Zhong Tang^*^*

**Synthetic routes to OTBP.** OTBP was synthesized by a two-step Suzuki coupling. In a 500-mL two-necked round bottom flask, 4,7-dibromobenzo[c]-1,2,5-thiadiazole (4 g, 13.61 mmol), 4-methoxy-*N*-(4-methoxyphenyl)-*N*-phenylaniline (4.28 g, 12.25 mmol), K_2_CO_3_ (5.64 g, 440.82 mmol), and Pd(PPh_3_)_4_ (471 mg, 0.41 mmol) were dissolved in a solution of THF/water (300 mL) with a ratio of 4:1 under the protection of nitrogen gas. The mixture was heated under reflux overnight at a stable temperature of 80 ℃ to produce OTBBr. After cooling to room temperature, the organic products were poured into water, extracted using DCM, and dried over anhydrous Na_2_SO_4_. The collected organic layer was then concentrated under reduced pressure. To isolate the main product OTBBr from the concentrated filtrate, flash silica gel column chromatography was performed using hexane/DCM as the eluent (2:1, v/v). The resulting product was used for the next step without further purification. The generated product was reacted with 4-pyridinylboronic acid (2.28 g, 18.5 mmol), Pd (PPh_3_)_4_ (700 mg, 0.62 mmol), and K_2_CO_3_ (2.56 g, 18.5 mmol) in a solution of THF/water (300 mL) with a ratio of 4:1 under the protection of nitrogen gas at 80 ℃ for 24 h. After cooling to room temperature, the organic products were extracted using DCM and dried over anhydrous Na_2_SO_4_. The collected organic layer was then concentrated under reduced pressure. The concentrated filtrate was purified by silica gel column chromatography using DCM/MeOH as the eluent (50:1, v/v) to obtain OTBP (3.3 g, 62.6%) as a red solid. ^1^H NMR (400 MHz, chloroform-*d*), *δ* ppm 8.81 - 8.75 (m, 2H), 8.26 - 8.20 (m, 2H), 7.97 (d, *J* = 7.5 Hz, 1H), 7.86 (d, *J* = 8.8 Hz, 2H), 7.79 (d, *J* = 7.5 Hz, 1H), 7.17 - 7.13 (m, 4H), 7.05 (d, J = 8.8 Hz, 2H), 6.90 - 6.86 (m, 4H), 3.82 (s, 6H). ^13^C NMR (101 MHz, CDCl_3_) *δ* ppm 156.57, 154.34, 153.85, 150.12, 149.63, 145.27, 140.54, 135.27, 130.15, 129.29, 128.93, 128.35, 127.44, 126.62, 123.72, 119.62, 115.05, 55.73. HRMS (ESI-TOF) for C_31_H_26_N_4_O_2_S [M+H]^+^: cald 517.1693, found 517.1694.

Scheme 1. Synthetic routes to OTBP

**Synthetic routes to ODBP.**

ODBP was synthesized by a Suzuki coupling reaction. ODBBr (1 g, 2.26 mmol) was reacted with 4-pyridinylboronic acid (834 mg, 6.78 mmol), Pd (PPh_3_)_4_ (260 mg, 0.2 mmol), and K_2_CO_3_ (938 mg, 6.78 mmol) in a solution of THF/water (300 mL) with a ratio of 4:1 under the protection of nitrogen gas at 80 ℃ for 24 h. After cooling to room temperature, the mixture was poured into water and extracted with DCM. The organic phase was combined and dried with anhydrous Na_2_SO_4_. The collected organic layer was then concentrated under reduced pressure. The concentrated filtrate was purified by silica gel column chromatography using DCM/MeOH as the eluent (50:1, v/v) to obtain ODBP as a deep red solid (253 mg, 25%). ^1^H NMR (400 MHz, chloroform-*d*), *δ* ppm 8.69 (d, *J* = 6.4 Hz, 2H), 8.06 (d, *J* = 6.4 Hz, 2H), 7.72 (d, *J* = 8.0 Hz, 1H), 7.07 - 7.02 (m, 4H), 6.99 (d, *J* = 8.0 Hz, 1H), 6.88 - 6.83 (m, 4H), 3.82 (s, 6H). ^13^C NMR (100 MHz, chloroform-*d*), *δ* (ppm) 157.02, 154.81, 150.43, 148.12, 147.15, 142.81, 141.02, 130.60, 126.86, 123.90, 123.50, 122.20, 118.47, 115.01, 55.80. HRMS (ESI-TOF) for C_25_H_22_N_4_O_2_S [M+H]^+^: cald 441.1380, found 441.1384.

Scheme 2. Synthetic routes to ODBP

**Density functional theory (DFT) calculations.** OTBP and ODBP were fully optimized with the DFT method using B3LYP density functional and 6-31G (d, p) basis set. Since the molecule was used in water or living cells, the polarizable continuum model (PCM) was used to consider the bulky solvation effects, with self-consistent reaction field (SCRF) and water as the solvent. London-dispersion effects were also considered using Grimme’s DFT-D3BJ correction to further describe long-range inter/intramolecular interactions. Time-dependent density functional theory (TD-DFT) was utilized at the same level of theory to calculate the transition dipole moment of OTBP and ODBP. Analytical frequency calculations were also performed at the same level of theory to confirm that the optimized structures were at a minimum point. All the above quantum chemical calculations were carried out using Gaussian 16 program. The frontier molecular orbitals (FMO) were displayed using IQmol molecular viewer package.

**Polarized optical microscope (POM).** The sample was placed on the carrier stage and a polarizer lens was inserted into the optical path. Bright-field shots were taken with the polarizer and analyzer in perpendicular directions. When taking polarized pictures, the angle between the polarizer and analyzer was adjusted by rotating the polarizer lens to obtain the best contrast and sharpness of the microscope image.

**Animal toxicity studies.** Healthy Balb/C mice aged 6–8 weeks were selected and divided into the control group and experimental group. The control group was intravenously injected with 100 μL of PBS every day, and the experimental group was intravenously injected with 100 μL of 1 mM solution of OTBP NCs every day. On the seventh day, the mice were put to death and the harvested organs were fixed. H&E staining was used to determine the damage of each organ.

**Calculations of BTHG imaging depth**. To further evaluate the ultimate imaging depth under objectives with larger working distance, we plot the relationship between the logarithm of pulse energy (*lg E*_in_) and imaging depth (*d*). The imaging depth in our system is defined as the depth at which the signal-to-noise ratio (SNR) drops below a threshold of 5. Importantly, the noise level in our system is relatively independent of imaging depth, and the decay of excitation intensity with depth can be described as *I*(*d*) *= I*_0_ *e* ^(^*^-μ d^*^)^, where *I*(*d*) is the excitation intensity at depth *d*, *I*_0_ is the initial intensity at the surface, and *μ* is the effective attenuation coefficient accounting for absorption and scattering. Taking the natural logarithm, we get *ln I*(*d*) = *ln I*_0_ - *μd.* Since the THG signal is proportional to the cube of the excitation intensity, the SNR at depth *d* depends on (*I*(*d*))*^3^*. Consequently, if we define the imaging depth as the point where the SNR reaches a constant threshold, the required excitation intensity at that depth also becomes constant. This transforms the problem of imaging depth into the form *d* = (*ln I*_0_ *- constant*) */μ*. Given that *E*_in_ determines *I*_0_, this equation indicates that *d* is directly proportional to *lg E*_in_. Our experimental results confirm this linear relationship, as shown in the plot of Figure 6e. Thus, increasing *E*_in_ can further extend the imaging depth, demonstrating the potential for deep imaging with our THG microscopy system and OTBP NCs. So, based on our experimental data, an imaging depth of 1.35 mm can be achieved when the pulse energy is increased to 20 nJ, which is the typical energy of three-photon deep-brain imaging. The calculation formula is shown as follows:

$$d (\mu m)=\left[ \left( \lg\left( E_{in} \right)-\lg{(E}_{300 \mu m}) \right)/Slope \right]+300$$

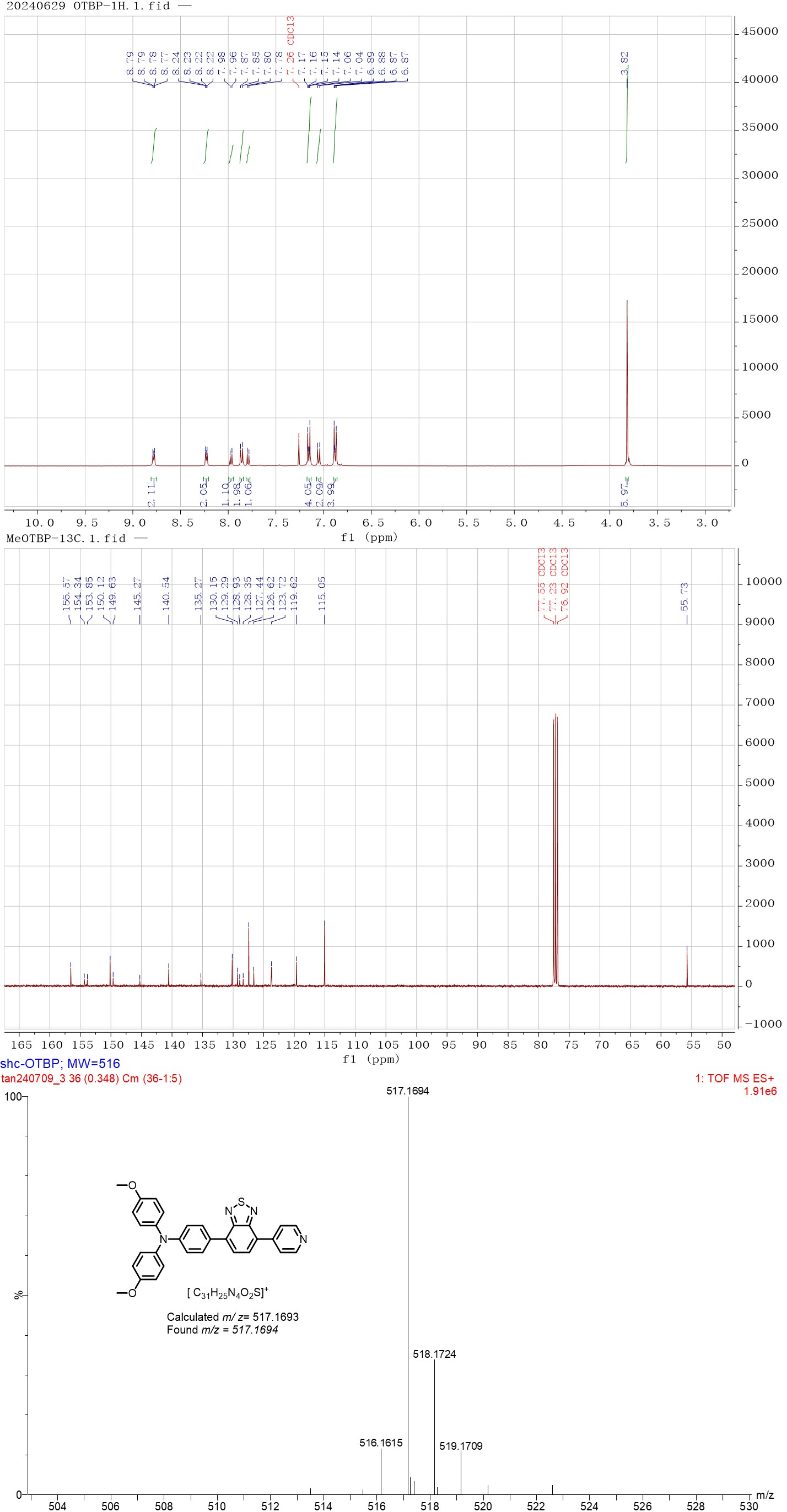


**Figure S1.** ^1^H NMR, ^13^C NMR and ESI-TOF-MS spectrum of OTBP.


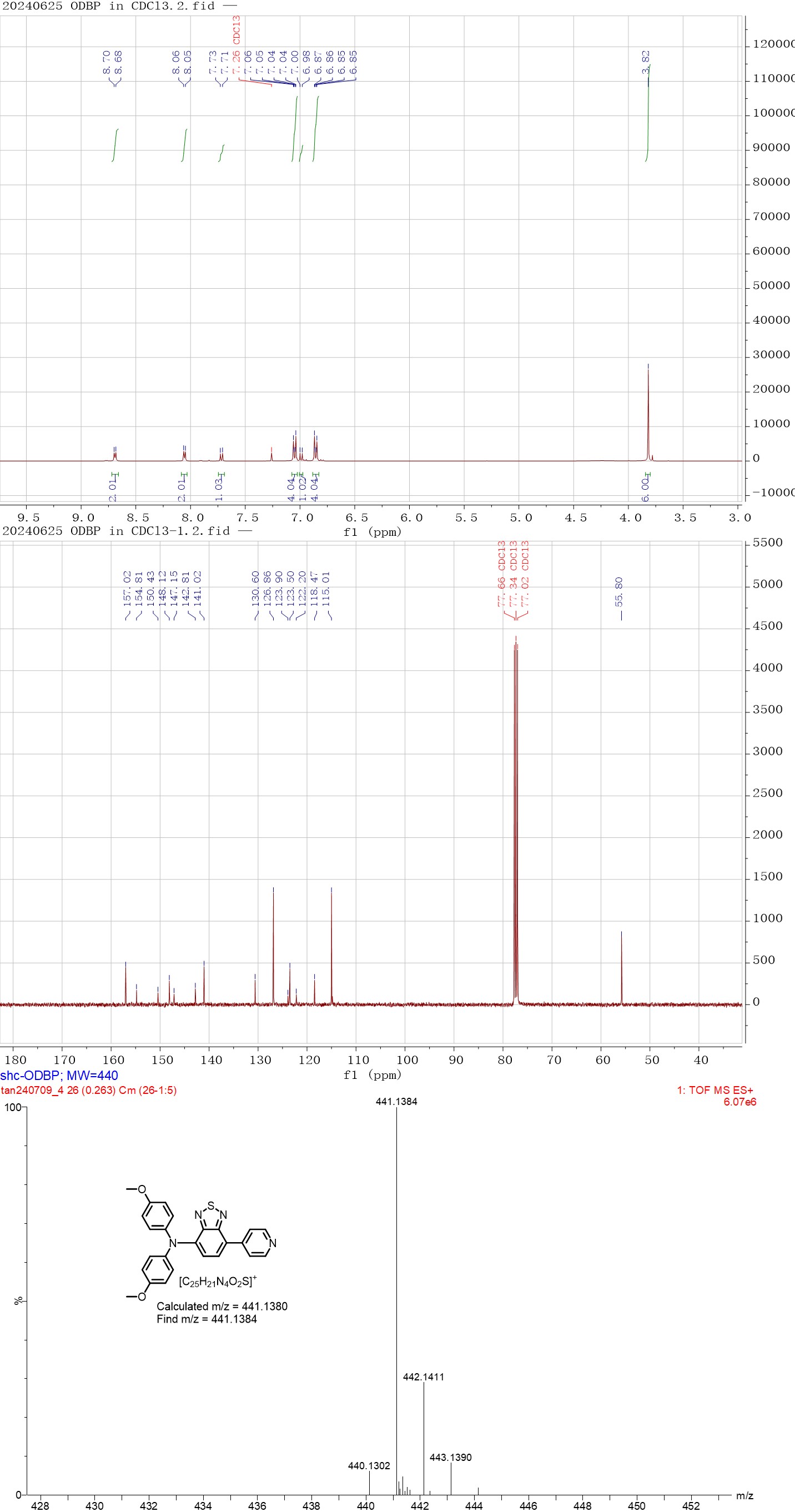


**Figure S2.** ^1^H NMR, ^13^C NMR and ESI-TOF-MS spectra of ODBP.


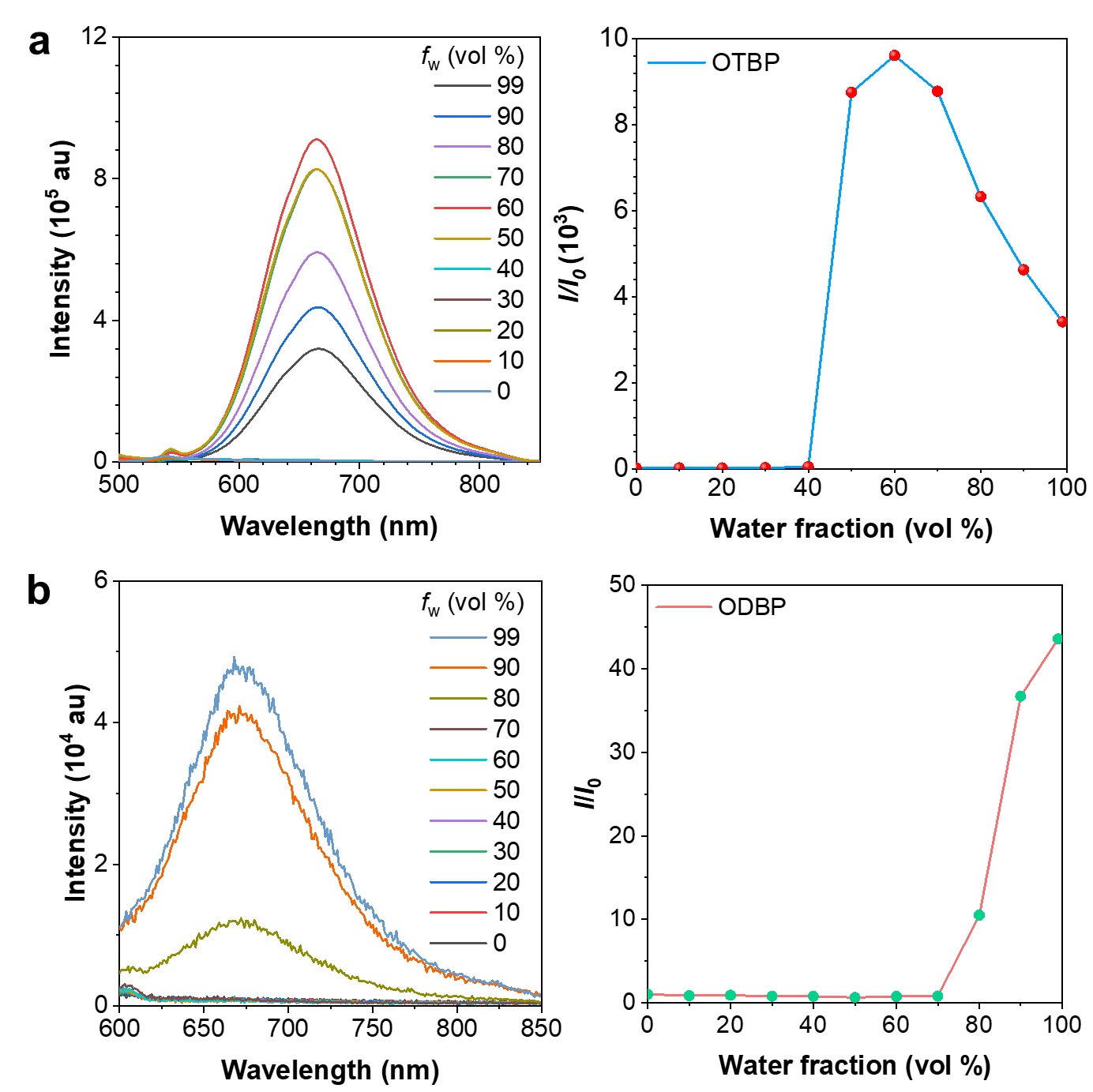


**Figure S3.** a) PL spectra of OTBP in DMSO/water mixtures with different water volume fraction (*f*_w_) and plot of relative PL intensity (*I/I*_0_) *versus* *f*_w_, where *I*_0_ = PL intensity in DMSO. [OTBP] = 10 μM; *λ*_ex_ = 470 nm. b) PL spectra of ODBP in DMSO/water mixtures with different water volume fraction (*f*_w_) and plot of relative PL intensity (*I/I*_0_) *versus* *f*_w_. [ODBP] = 10 μM; *λ*_ex_ = 520 nm.


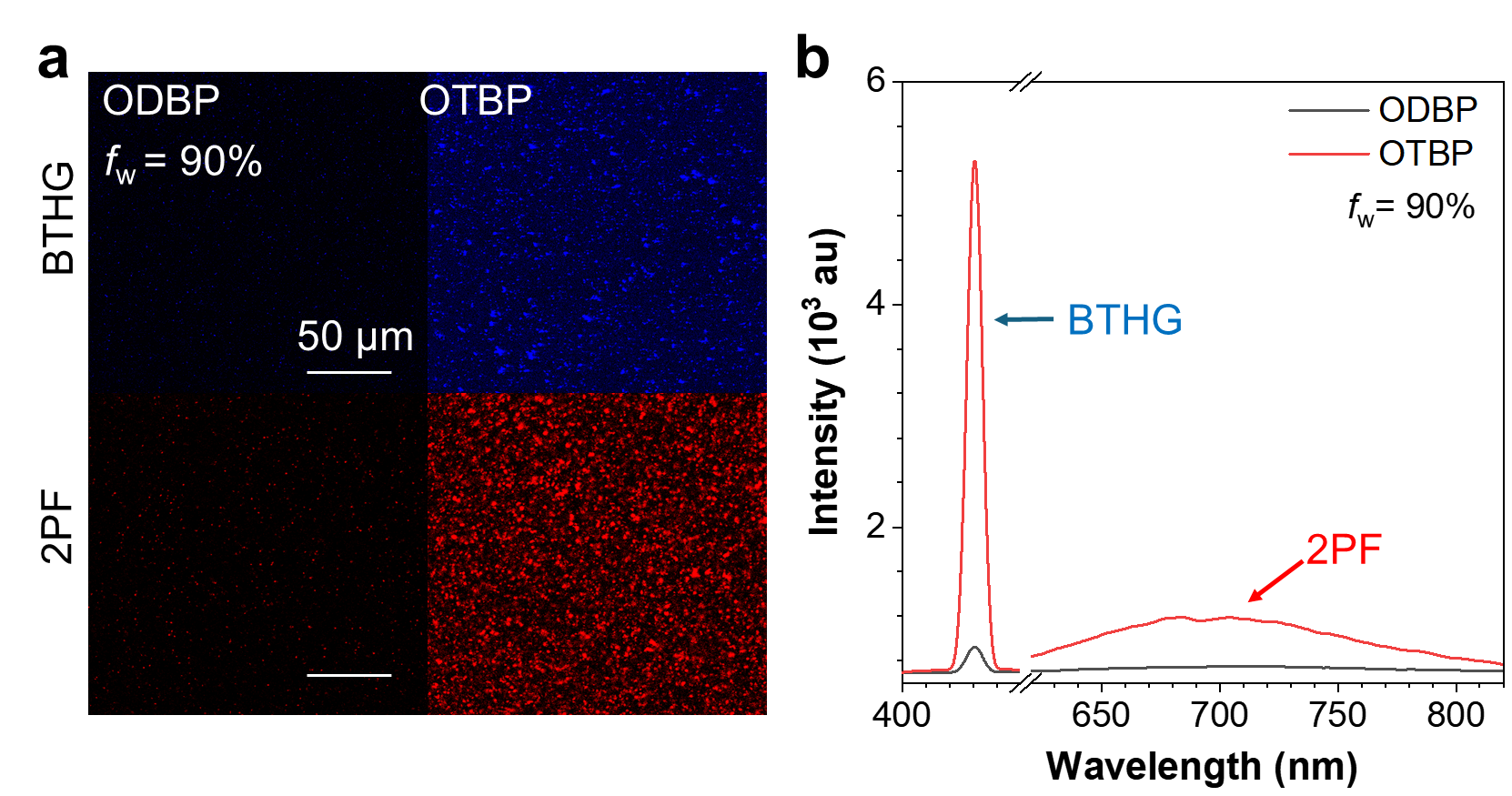


**Figure S4.** a**)** BTHG and 2PF images of OTBP and ODBP aggregates formed in a DMSO/water mixture at *f*_w_ = 90%. *λ*_ex_ = 1040 nm and *λ*_em_ = 570–750 nm for 2PF; *λ*_ex_ = 1300 nm and *λ*_em_ = 425–475nm for BTHG; [OTBP] = 100 μM. b) BTHG and 2PF spectra of OTBP and ODBP aggregates formed in a DMSO/water mixture at *f*_w_ = 90%.


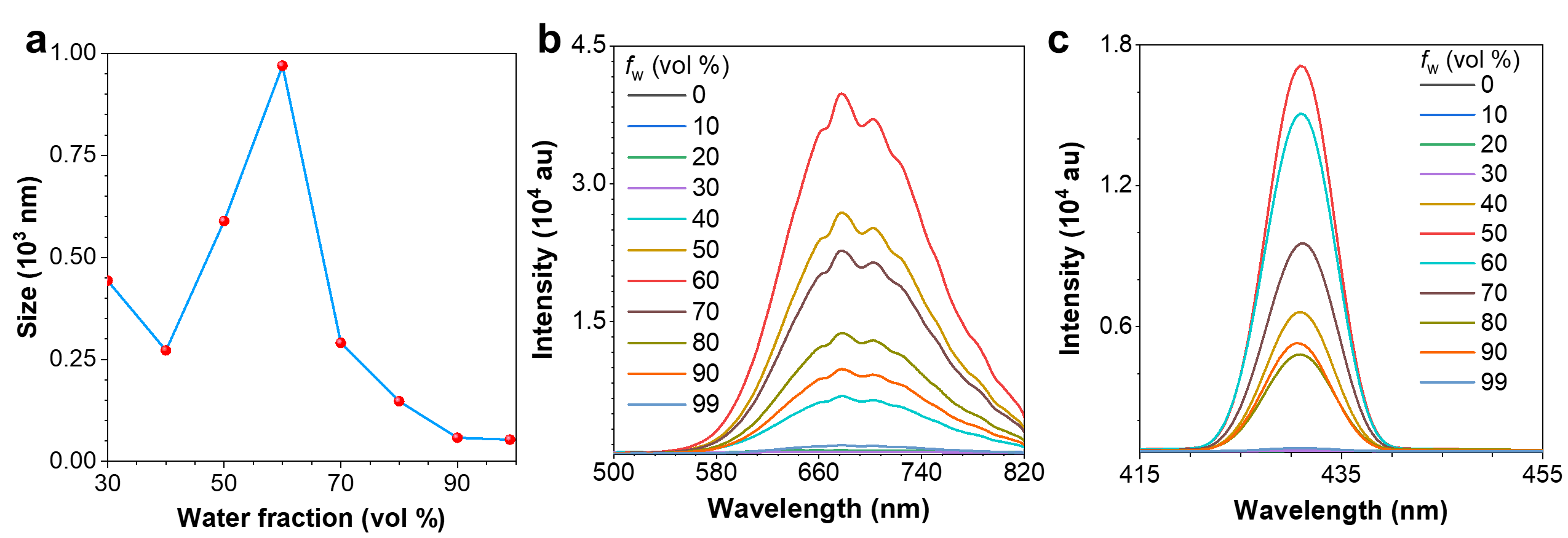


**Figure S5.** a) Plot of particle size of OTBP aggregates *versus* *f*_w_. b) 2PF spectra of OTBP in DMSO/water mixtures with different *f*_w_. *λ*_ex_ = 1040 nm; [OTBP] = 100 μM. c) BTHG spectra of OTBP at different *f*_w_. *λ*_ex_ = 1300 nm.


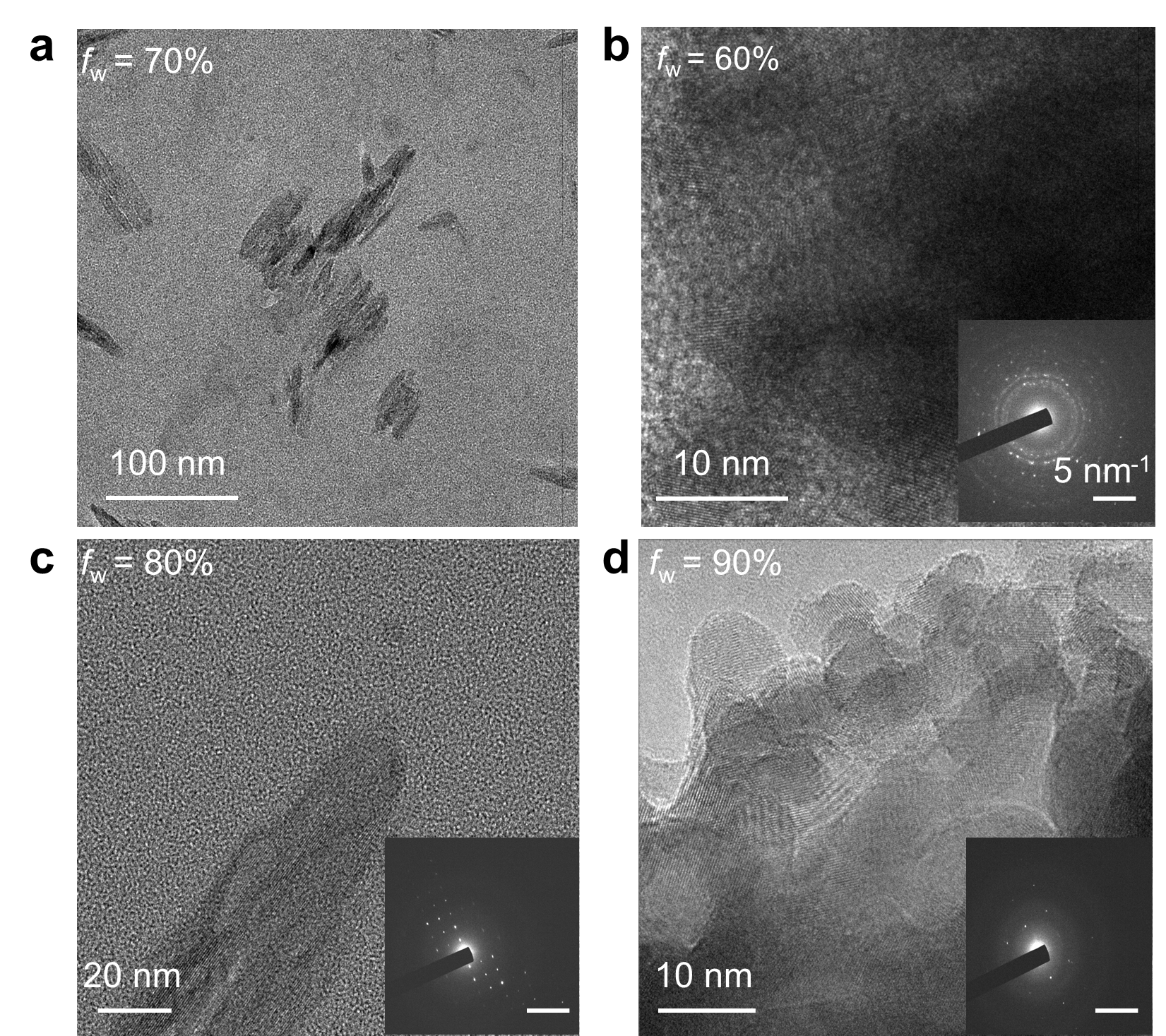


**Figure S6.** a) TEM image of OTBP aggregates formed in a DMSO/water mixture at *f_w_* = 70%. b–d) TEM image and selected area electron diffraction pattern of OTBP aggregates formed in a DMSO/water mixture at different *f*_w_. Inset: selected area electron diffraction pattern of corresponding sample.


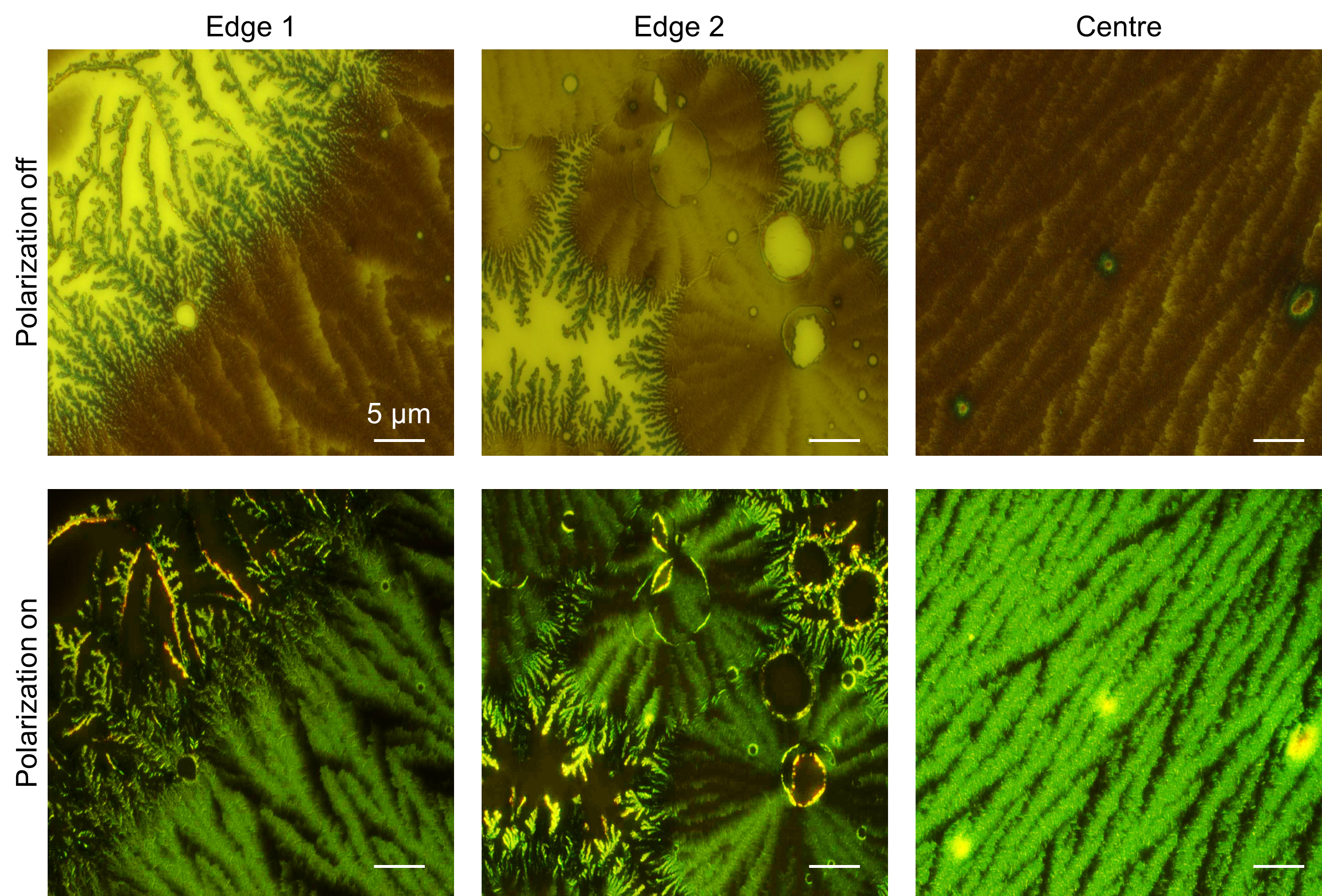


**Figure S7.** Optical images of the obtained OTBP film under polarized optical microscope with polarization on or off. The images reveal the edge and central structures of the film.


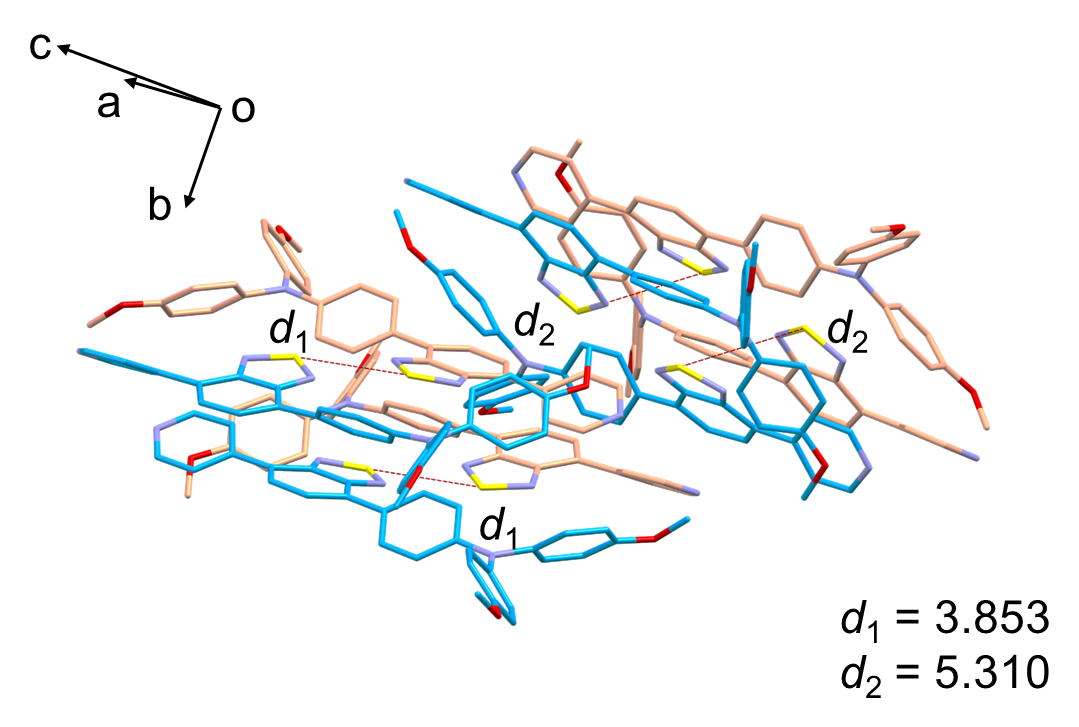


**Figure S8.** Molecular packing of OTBP in the interlayer.


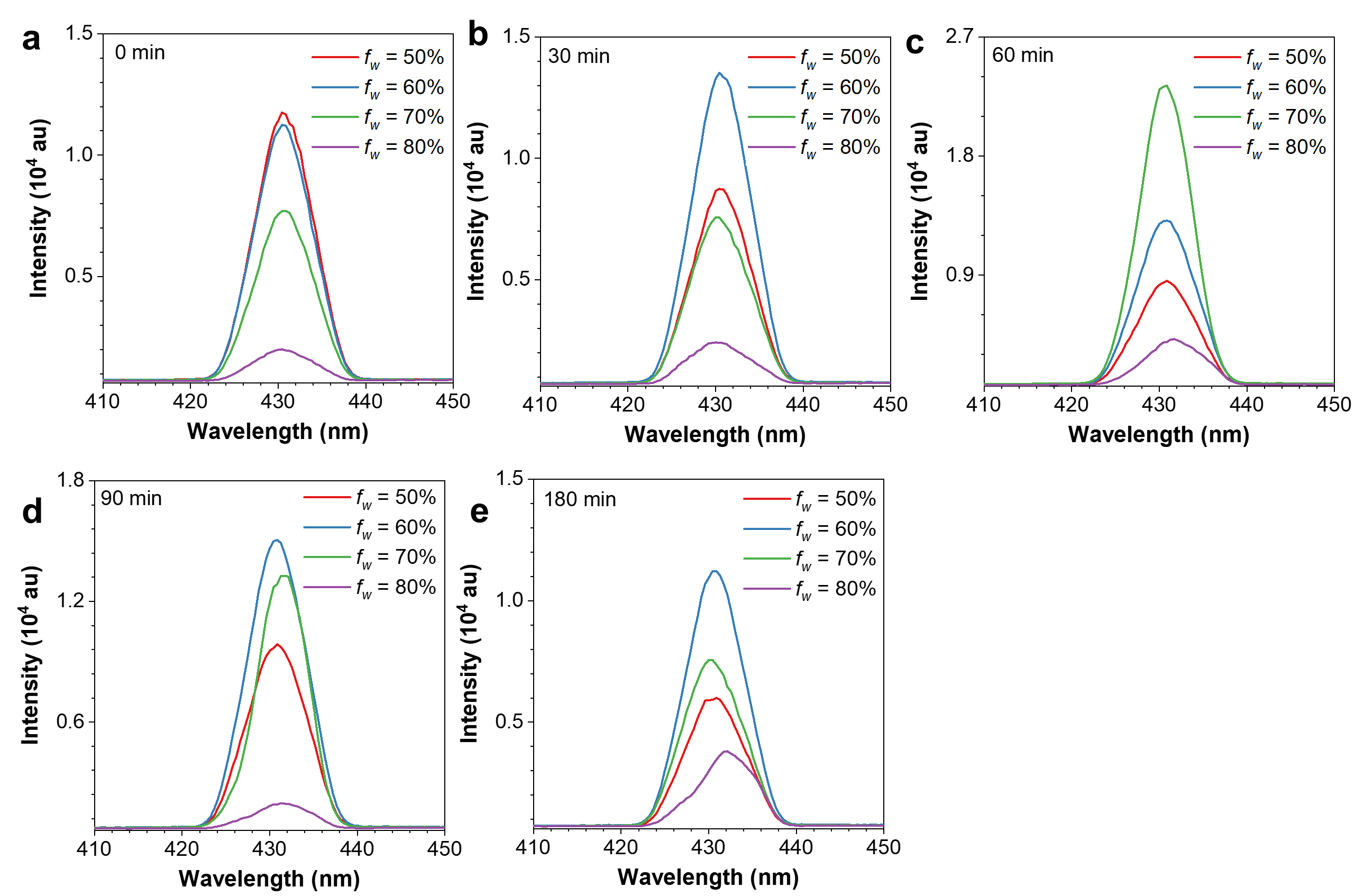


**Figure S9.** BTHG spectral changes of OTBP NCs formed in the DMSO/water mixture at different *f*_w_ with aging time (0–180 min), *λ*_ex_ = 1300 nm.


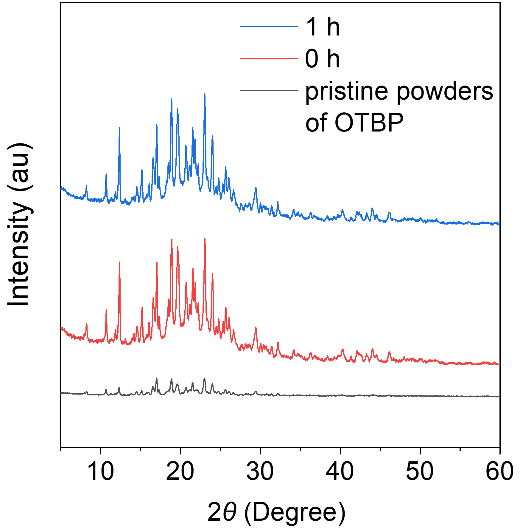


**Figure S10.** PXRD spectra of OTBP pristine powders and NCs formed in the DMSO/water mixture at *f*_w_ = 70% after aging for 0 h or 1 h.


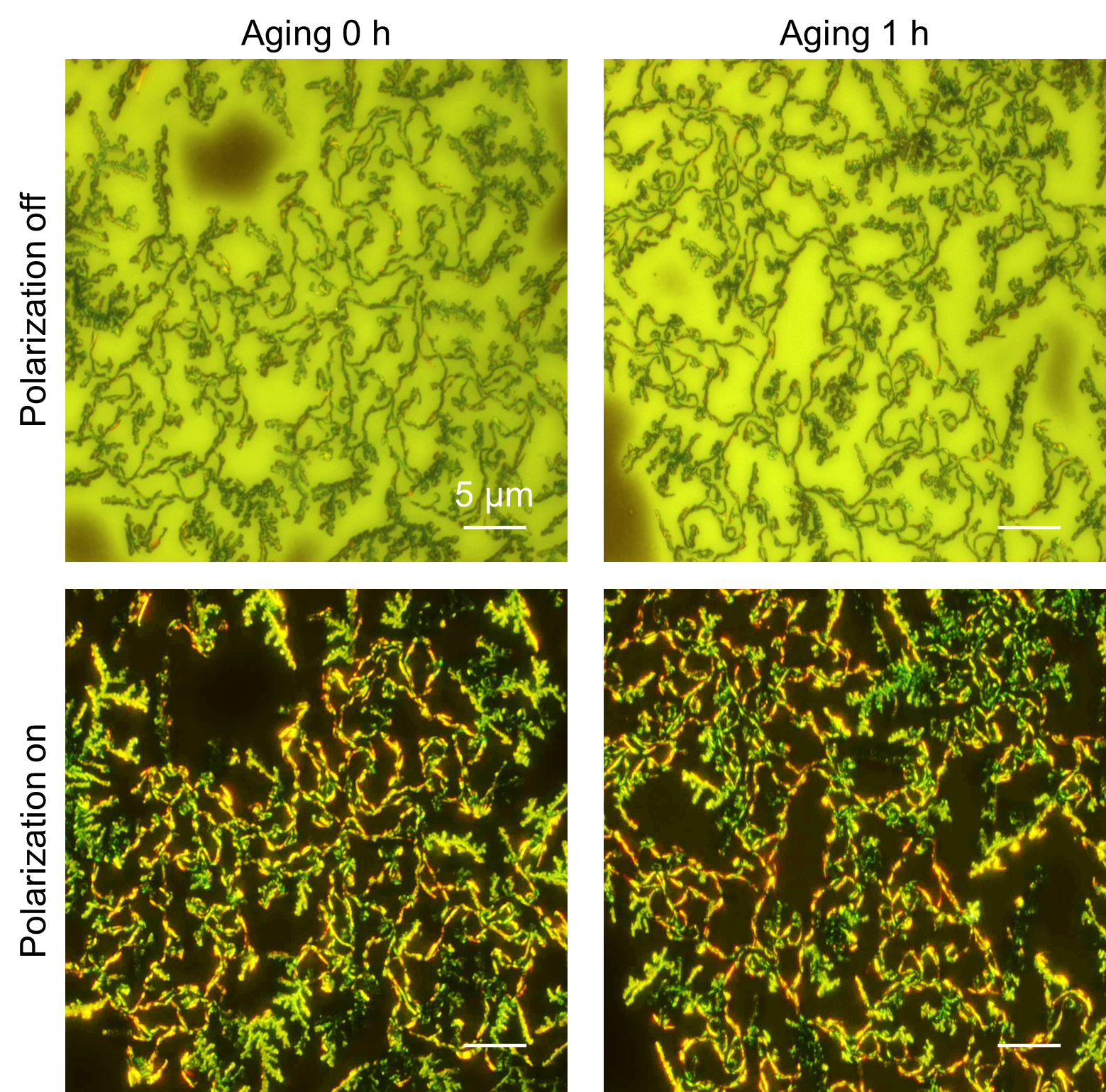


**Figure S11.** Polarization-on and polarization-off optical images of OTBP NCs formed in the DMSO/water mixture at *f*_w_ = 70% after aging for 0 h or 1 h.


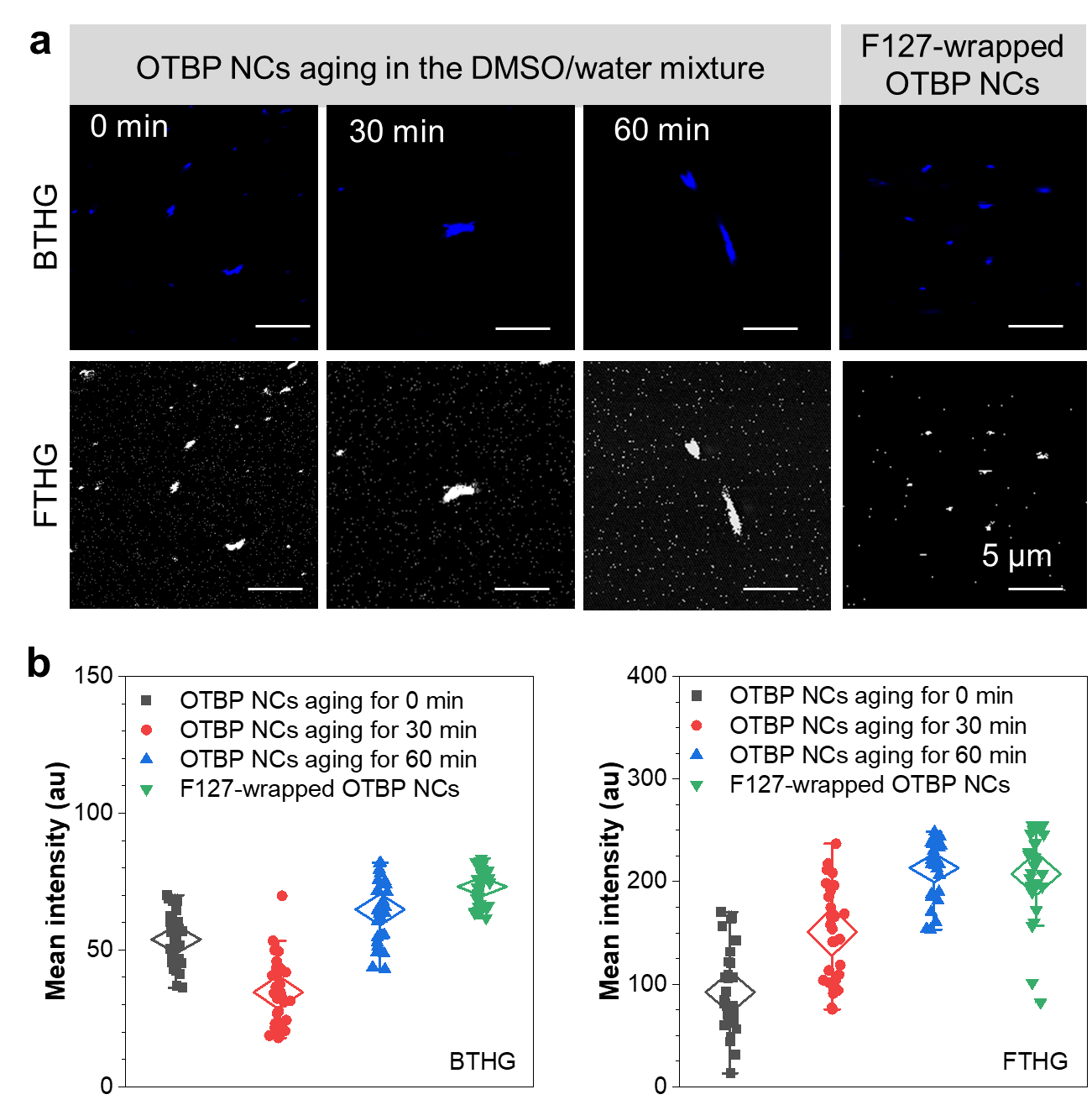


**Figure S12.** a) BTHG and FTHG images of OTBP NCs with different aging times in the DMSO/water mixture (*f_w_* = 70%) and F127-wrapped OTBP NCs. b) BTHG and FTHG mean intensity of OTBP NCs with different aging times in the DMSO/water mixture (*f_w_* = 70%) and F127-wrapped OTBP NCs. 30 particles of each sample were selected for statistical analysis of THG mean intensity.


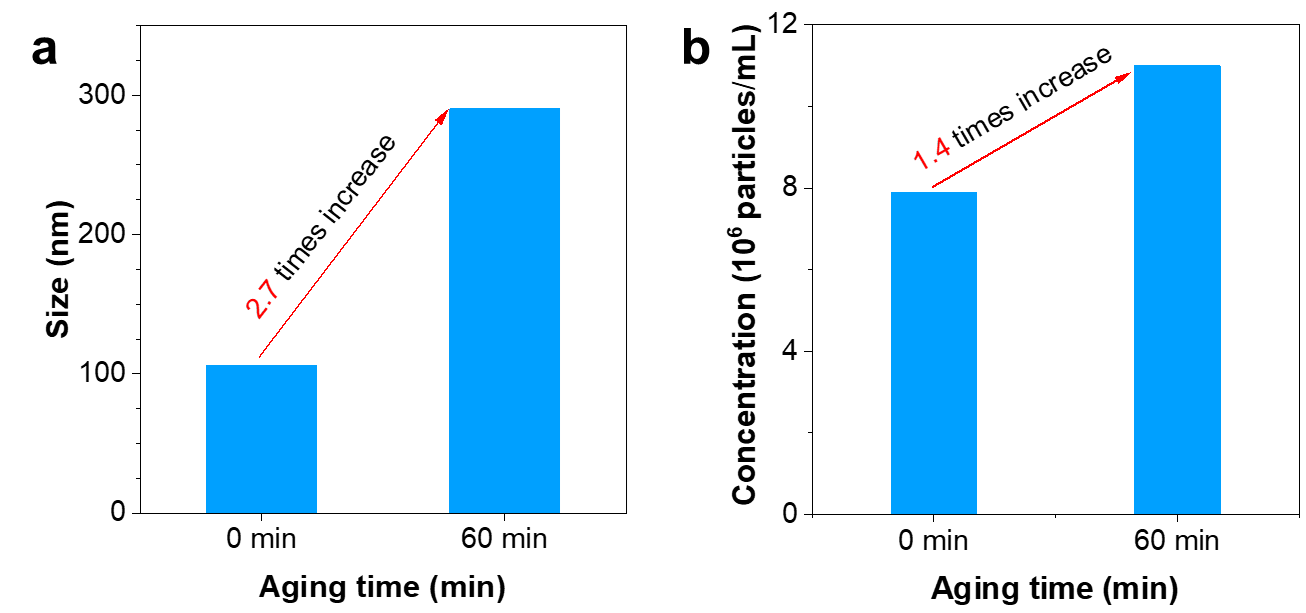


**Figure S13.** a) Size change of OTBP NCs when aging in the DMSO/water mixture (*f_w_* = 70%). b) Particle concentration change with aging time.


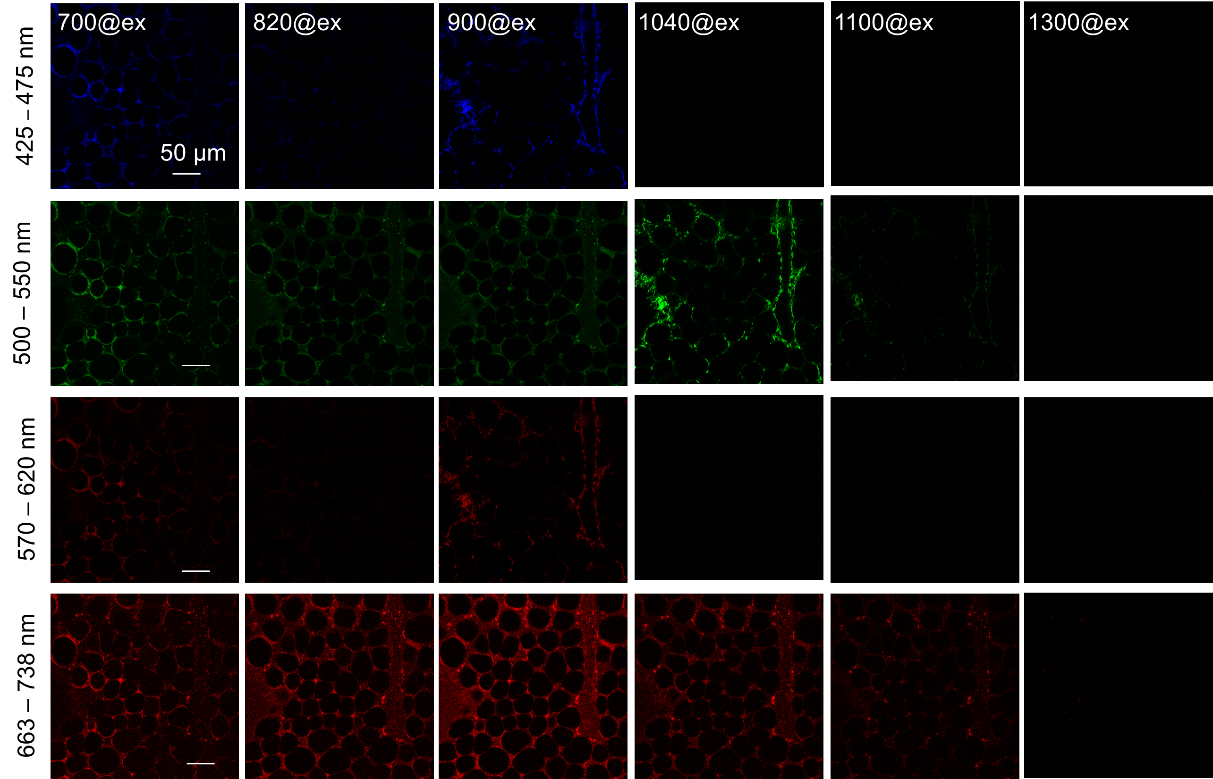


**Figure S14.** Autofluorescence images of *ex vivo* intestine slices at various excitation wavelengths. *λ*_em_ = 425–475 nm, 500–550 nm, 570–620 nm, and 663–738 nm.


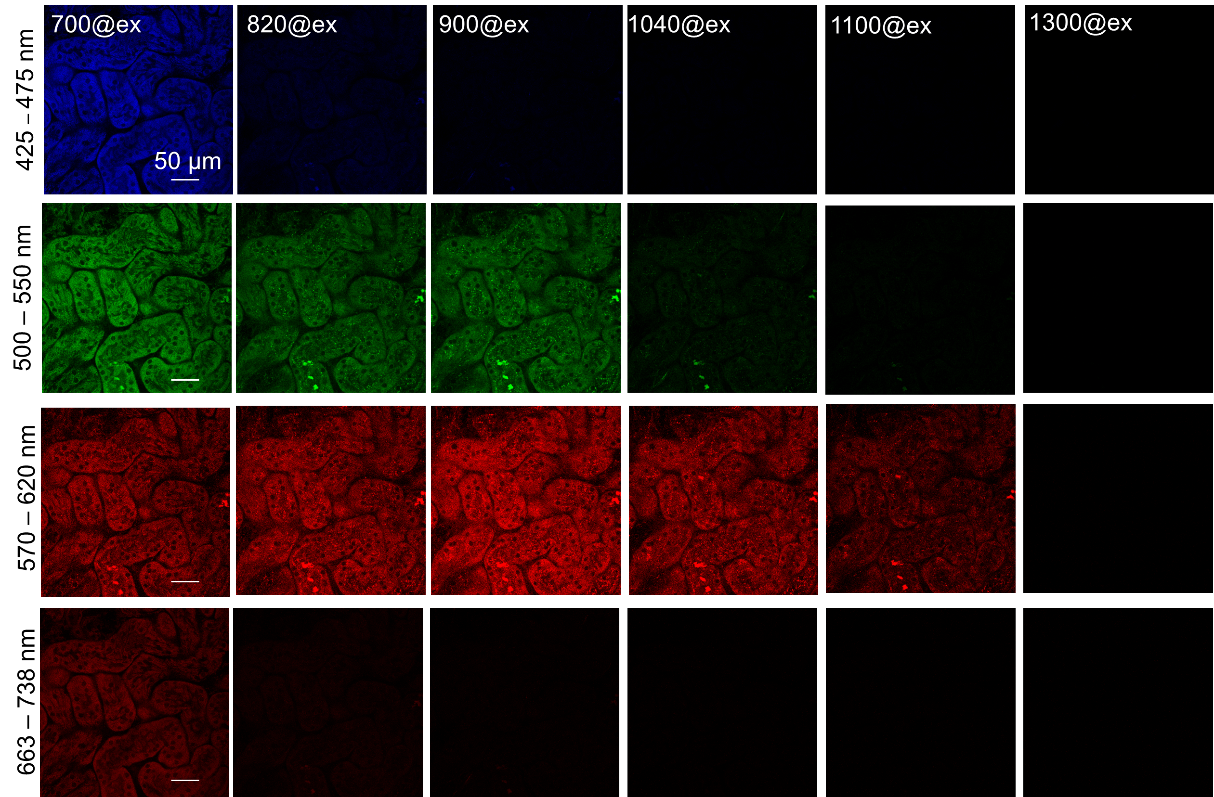


**Figure S15.** Autofluorescence images of *ex vivo* kidney slices at various excitation wavelengths. *λ*_em_ = 425–475 nm, 500–550 nm, 570–620 nm, and 663–738 nm.


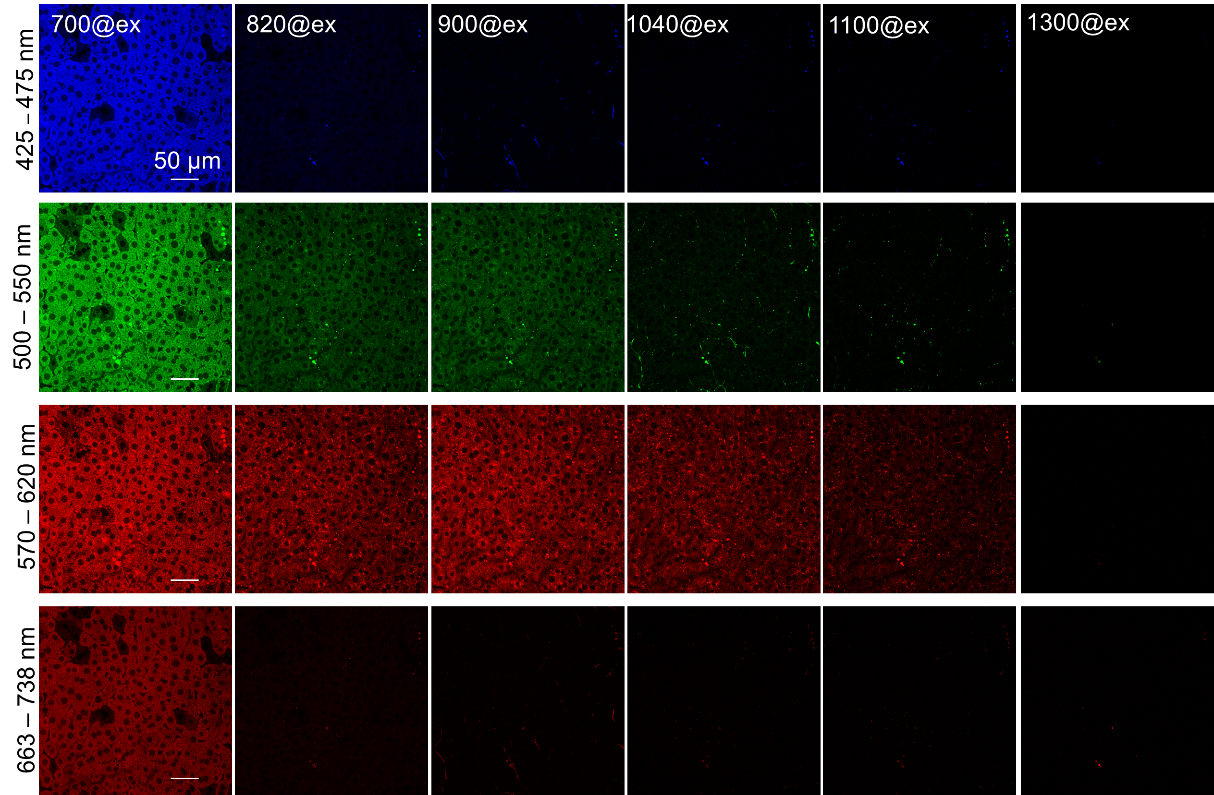


**Figure S16.** Autofluorescence images of *ex vivo* liver slices at various excitation wavelengths. *λ*_em_ = 425–475 nm, 500–550 nm, 570–620 nm, and 663–738 nm.


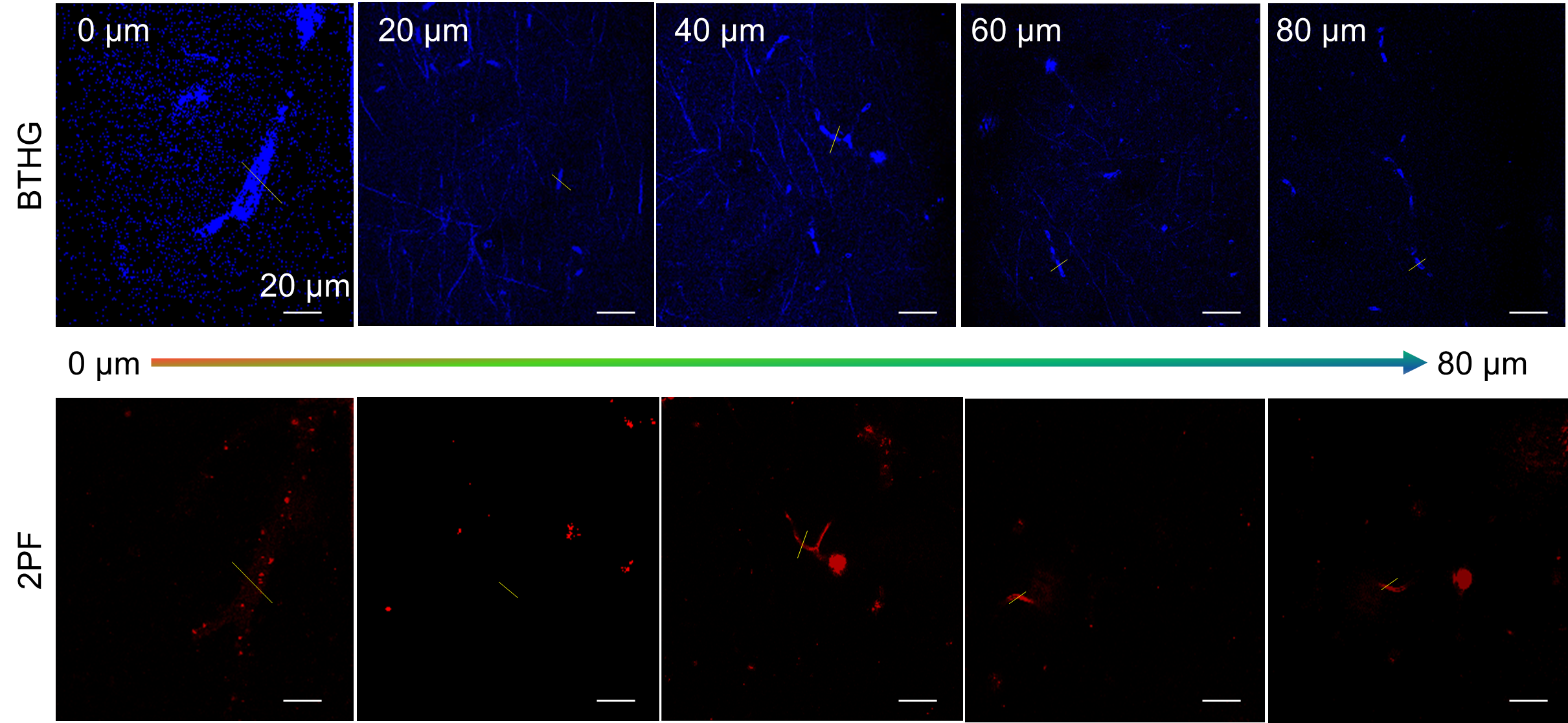


**Figure S17.** BTHG and 2PF images of mouse brain vessels at different depths post intravenous injection of OTBP NCs into mice. *λ*_ex_ = 1260 nm for BTHG; *λ*_ex_ = 1040 nm for 2PF; *P* = 15 mW (~200-pJ).


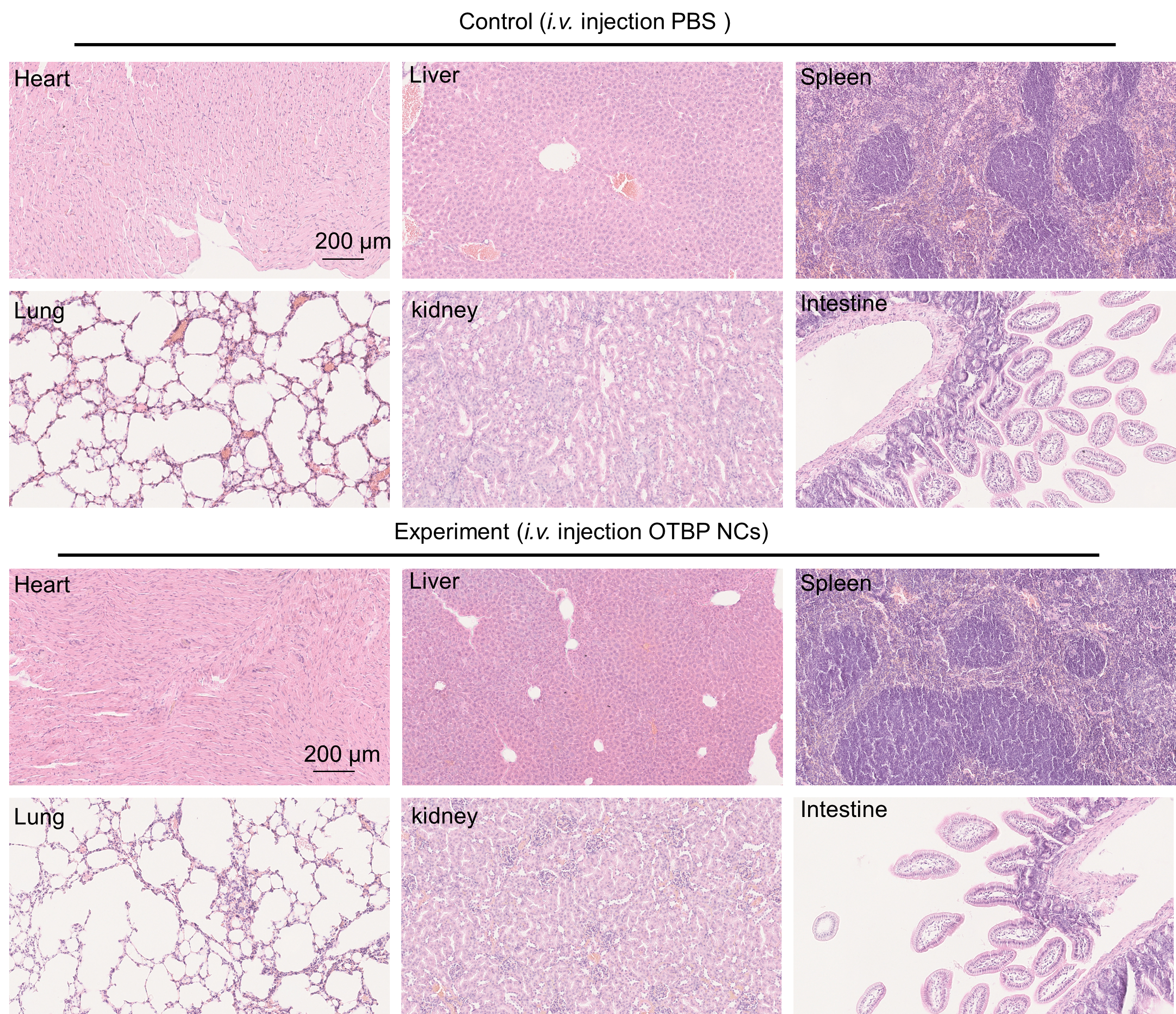


**Figure S18.** H&E staining of heart, liver, spleen, lung, kidney, and intestine. Mice were injected with OTBP NCs (1 mM, 100 μL) via tail vein, while the control group received an equal volume of PBS.

**Table S1.** Crystal data and structure refinement for OTBP.

| Identification code | OTBP |
| --- | --- |
| Empirical formula | C_31_H_24_N_4_O_2_S |
| Formula weight | 516.60 |
| Temperature/K | 173.00(10) |
| Crystal system | monoclinic |
| Space group | P2_1_/c |
| a/Å | 21.35809(11) |
| b/Å | 12.69064(7) |
| c/Å | 18.63137(9) |
| α/° | 90 |
| β/° | 93.3685(5) |
| γ/° | 90 |
| Volume/Å^3^ | 5041.26(5) |
| Z | 8 |
| ρ_calc_g/cm^3^ | 1.361 |
| μ/mm^-1^ | 1.440 |
| F(000) | 2160.0 |
| Crystal size/mm^3^ | 0.15 × 0.12 × 0.03 |
| Radiation | Cu Kα (*λ* = 1.54184) |
| 2*Θ* range for data collection/° | 8.108 to 148.696 |
| Index ranges | -23 ≤ h ≤ 26, -15 ≤ k ≤ 11, -22 ≤ l ≤ 21 |
| Reflections collected | 30324 |
| Independent reflections | 10080 [R_int_ = 0.0159, R_sigma_ = 0.0154] |
| Data/restraints/parameters | 10080/0/689 |
| Goodness-of-fit on F^2^ | 1.034 |
| Final R indexes [I>=2σ (I)] | R_1_ = 0.0319, wR_2_ = 0.0851 |
| Final R indexes [all data] | R_1_ = 0.0350, wR_2_ = 0.0879 |
| Largest diff. peak/hole / e Å^-3^ | 0.23/-0.34 |
